# A *Penicillium chrysogenum*-based expression system for the production of small, cysteine-rich antifungal proteins for structural and functional analyses

**DOI:** 10.1186/s12934-016-0586-4

**Published:** 2016-11-11

**Authors:** Christoph Sonderegger, László Galgóczy, Sandra Garrigues, Ádám Fizil, Attila Borics, Paloma Manzanares, Nikoletta Hegedüs, Anna Huber, Jose F. Marcos, Gyula Batta, Florentine Marx

**Affiliations:** 1 Division of Molecular Biology, Biocenter, Medical University of Innsbruck, Innrain 80-82, Innsbruck, 6020 Austria; 2 Biotechnology Department, Instituto de Agroquímica y Tecnología de Alimentos (IATA), Consejo Superior de Investigaciones Científicas (CSIC), Avenida Agustín Escardino 7, 46980 Paterna, Valencia Spain; 3 Department of Organic Chemistry, Faculty of Science and Technology, University of Debrecen, Egyetem tér 1, 4032 Debrecen, Hungary; 4 Institute of Biochemistry, Biological Research Centre of Hungarian Academy of Sciences, Temesvári krt. 62, 6726 Szeged, Hungary; 5 Sandoz GmbH, Biochemiestrasse 10, 6250 Kundl, Austria

**Keywords:** Antifungal proteins, PAF, NFAP, *Penicillium chrysogenum*, *Penicillium digitatum*, *Neosartorya fischeri*, Recombinant protein production, Electronic circular dichroism (ECD) spectroscopy, Nuclear magnetic resonance (NMR)

## Abstract

**Background:**

Small, cysteine-rich and cationic antifungal proteins (APs) from filamentous ascomycetes, such as NFAP from *Neosartorya fischeri* and PAF from *Penicillium chrysogenum,* are promising candidates for novel drug development. A prerequisite for their application is a detailed knowledge about their structure–function relation and mode of action, which would allow protein modelling to enhance their toxicity and specificity. Technologies for structure analyses, such as electronic circular dichroism (ECD) or NMR spectroscopy, require highly purified samples and in case of NMR milligrams of uniformly ^15^N-/^13^C-isotope labelled protein. To meet these requirements, we developed a *P. chrysogenum*-based expression system that ensures sufficient amount and optimal purity of APs for structural and functional analyses.

**Results:**

The APs PAF, PAF mutants and NFAP were expressed in a *P. chrysogenum* ∆*paf* mutant strain that served as perfect microbial expression factory. This strain lacks the *paf*-gene coding for the endogenous antifungal PAF and is resistant towards several APs from other ascomycetes. The expression of the recombinant proteins was under the regulation of the strong *paf* promoter, and the presence of a *paf*-specific pre-pro sequence warranted the secretion of processed proteins into the supernatant. The use of defined minimal medium allowed a single-step purification of the recombinant proteins. The expression system could be extended to express PAF in the related fungus *Penicillium digitatum*, which does not produce detectable amounts of APs, demonstrating the versatility of the approach. The molecular masses, folded structures and antifungal activity of the recombinant proteins were analysed by ESI–MS, ECD and NMR spectroscopy and growth inhibition assays.

**Conclusion:**

This study demonstrates the implementation of a *paf* promoter driven expression cassettes for the production of cysteine-rich, cationic, APs in different *Penicillium* species. The system is a perfect tool for the generation of correctly folded proteins with high quality for structure–function analyses.

**Electronic supplementary material:**

The online version of this article (doi:10.1186/s12934-016-0586-4) contains supplementary material, which is available to authorized users.

## Background

Antifungal proteins (APs) from filamentous ascomycetes are small in size, cysteine-rich and cationic. These pre-pro proteins are synthesized in the ribosome and processed to mature forms when secreted into the fungal culture broth [1]. Extensively studied examples are PAF from *Penicillium chrysogenum* [2], NFAP from *Neosartorya fischeri* [3–5] and AFP from *Aspergillus giganteus* [6]. A significant diversity of PAF related genes and proteins has been found in ascomycetes, with genomes encoding up to three different sequence-related AP groups that provide a rich source of potentially divergent antifungals [7]. These natural proteins inhibit the growth of human-, animal- and plant-pathogenic moulds [8]. No detrimental effects on plant or on mammalian cells in vitro and in vivo could be observed for PAF [9, 10] and AFP [11, 12]. These findings strongly support their applicability as new antifungal drugs or their use for the development of new antifungal strategies.

Detailed structure–function analyses, however, are indispensable for a potential improvement of activity and specificity by rational design and for any future application. To this end, high quality protein preparations in considerable amounts are required. However, the bio-molecules are mostly expressed in small quantities by the producing moulds. Recombinant techniques for over-expression of APs in heterologous systems encounter major problems: correct processing and disulphide bond formation are essential for full protein activity [13]. Gene over-expression in microbial cells is still a challenging issue. Protein production by *Escherichia coli* offers some advantages for its easy and cost-effective cultivation and high protein yields [14]. However, this expression system also exhibits some disadvantages: (1) codon bias when expressing eukaryotic genes; (2) endotoxin contamination of the protein preparation; (3) incorrect folding and disulphide bridge formation, and low solubility of proteins can lead to inclusion body formation, which complicates purification and makes protein refolding necessary [14, 15].

A more reliable expression system is the yeast *Pichia pastoris*; it copes with disulphide bond formation, glycosylation and proper protein processing and folding [14]. However, the *P. pastoris* expression system bears the risk of unwanted protein modifications, such as O-linked and non-covalently linked sugars. Furthermore, *P. pastoris* secretes high concentrations of mannan into the expression medium [16]. Such carbohydrates need to be extensively removed otherwise they are detrimental for NMR studies and limit the amount of structural information [17].

Filamentous ascomycetes, finally, have been developed for homologous and heterologous gene expression [18, 19]. *P. chrysogenum* has been successfully used as expression system applying inducible (xylanase *xylA*) or constitutive (NADP-dependent glutamate dehydrogenase *gdhA*) promoters [20, 21] and efforts were undertaken to define new promoters for strain engineering in *Aspergillus* and *Penicillium* spp. [22, 23].

**Fig. 1:**
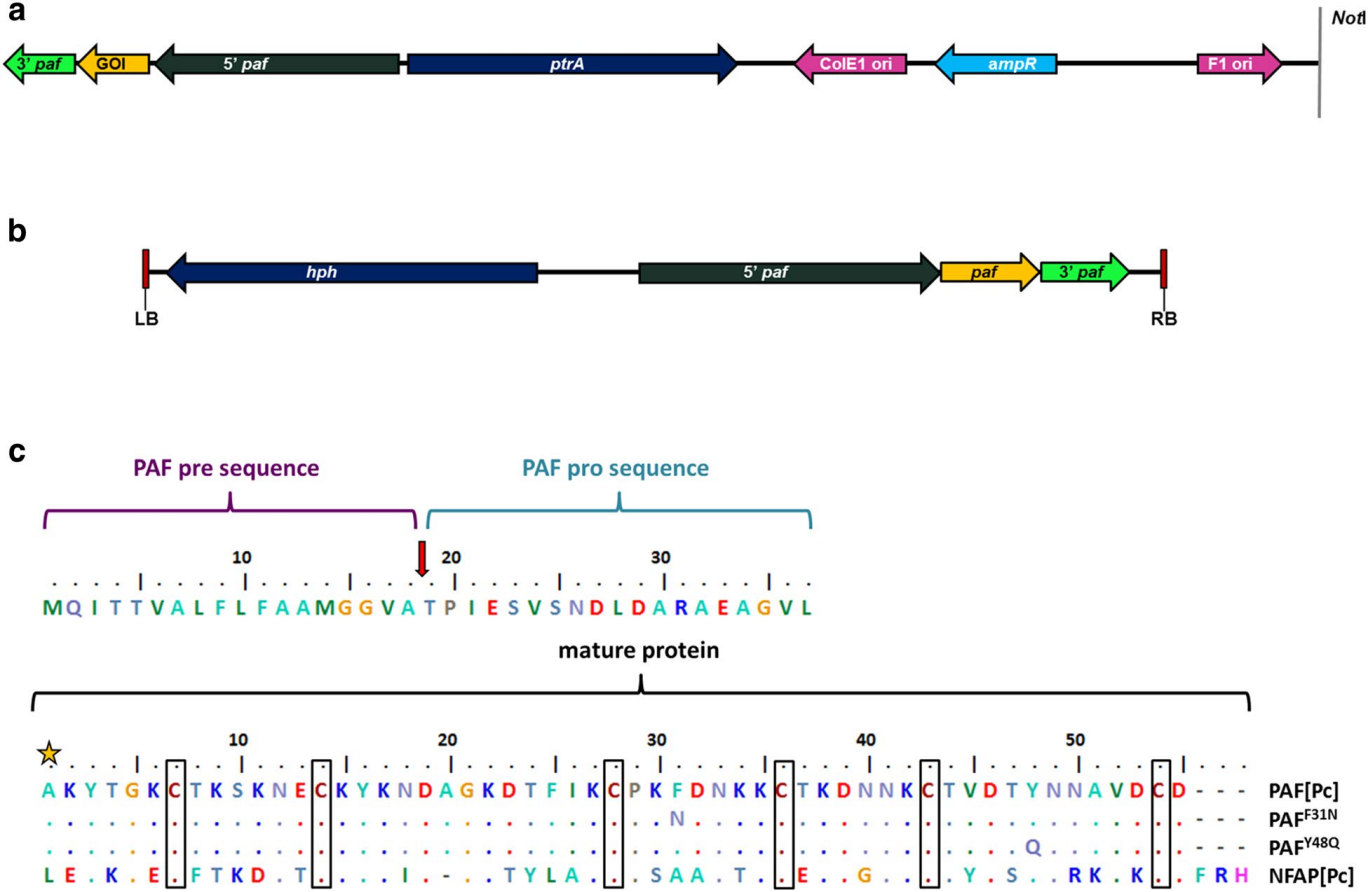
Schematic representation of the linearized expression plasmids **a** pSK275*paf* for the transformation in *P. chrysogenum* and **b** the T-DNA region of pBHt2_PAF binary vector for the *A. tumefaciens* mediated transformation of *P. digitatu*m. Colour code of the expression cassette: black, *paf* 5′ UTR (promoter); green, *paf* 3′ UTR (terminator); yellow, gene of interest to be expressed (pre-pro sequence included); **c** ClustalW (BioEdit) alignment of the pre-pro proteins PAF[Pc] (equivalent to PAF[Pd]), PAF^F31N^, PAF^Y48Q^ and NFAP[Pc]. *Top*: PAF-specific pre-pro sequence; *Bottom*: mature proteins. The amino acid positions are numbered. Distinct amino acids are represented in colour code, identical amino acids are aligned in *coloured dots*, cysteine residues are *boxed*. The *arrow* indicates the predicted signal sequence cleavage site, the first amino acid of the mature proteins is marked with an *asterisk*. *GOI* gene of interest, *hph* hygromycin resistance gene cassette, *ptrA* pyrithiamine resistance gene cassette, *ampR* ampicillin resistance gene cassette, *LB/RB* left/right borders, F1 ori single-stranded phagemid origin; *ColE1* origin, plasmid origin of replication, *Not*I restriction site for linearization of pSK275*paf*

In this study we provide a new and important example for the appropriateness of *Penicillium* spp. as expression systems. We present an expression cassette consisting of the strong *paf* gene promoter, the *paf* pre-pro sequence for correct protein processing and secretion and the *paf* gene termination signal [24]. This expression cassette was used in *P. chrysogenum* for the production of high yields of recombinant, cysteine-rich APs for structural and functional analyses. The cultivation of the genetically engineered *P. chrysogenum* strains in defined minimal medium allowed an easy, single-step chromatographic purification of the proteins from the culture broth. With this expression system we generated PAF and PAF mutants with amino acid exchanges to investigate the role of specific protein motifs in antifungal function. Moreover, we used this system for the heterologous expression of the aforementioned AP NFAP from *N. fischeri*, which was found to be harmless for *P. chrysogenum* (unpublished data). Finally, we also tested the applicability of the *P. chrysogenum* expression cassette in another *Penicillium* species: we generated PAF in the post-harvest phyto-pathogenic fungus *Penicillium digitatum* that does not produce detectable amounts of APs [7] and is tolerant to PAF (unpublished data). The mature APs PAF and NFAP are 55 and 57 amino acids long and contain six cysteine-residues (Fig. 1). In PAF, these cysteines form three disulphide bonds in *abcabc* pattern, which is essential for proper folding and full antifungal activity [13, 25, 26]. A similar folding is highly probable for NFAP [4]. Both proteins do not undergo any posttranslational modifications, except for cleavage of the pre-pro sequence and protein folding (Fig. 1).

Since not only the protein amount, but also the correct processing and folding is important [27], we paid special attention to characterize these features of recombinant PAF, PAF mutants and NFAP by using electro-spray ionization mass spectrometry (ESI–MS), electronic circular dichroism (ECD) and nuclear magnetic resonance (NMR) spectroscopy. The activity of the recombinant APs was determined in broth microdilution assays with *Aspergillus niger* as test organism.

## Results

### Protein expression and purification

The expression cassette consisted of the *paf* gene (420 bp) flanked by approximately 1280 bp of the 5′-UTR and 370 bp of the 3′-UTR and was inserted into plasmid pSK275 that contains the pyrithiamine resistance gene (*ptrA*) for selection of positive *P. chrysogenum* transformants and the ampicillin resistance gene (*amp*) for plasmid propagation in *E. coli* [28] (Fig. 1a). This plasmid pSK275*paf* was used to over-express PAF (PAF[Pc]) and served as basis for all further constructs (Additional File 1: Fig. S1).

The plasmid pSK275*paf* was used as a template for PCR-based site-directed mutagenesis to mutate distinct codons of the *paf* gene for amino acid substitutions and the production of PAF mutants. The preferential codon usage of *P. chrysogenum* was taken into account for gene modification. The amino acids Phe25, Ile26, Phe31 and Tyr48 form a hydrophobic patch on the surface of PAF [13, 26]. This motif is assumed to play a major role in the interaction of this AP with the plasma membrane of target fungi [13, 26]. To address this assumption, we exchanged Phe31 and Tyr48 with the uncharged, polar residues asparagine and glutamine, respectively and created PAF^F31N^ and PAF^Y48Q^ (Fig. 1c).

We further used the *P. chrysogenum*-based expression system to increase the yield and purity of the *N. fischeri* AP NFAP for structural analyses. To this end, the *nfap* cDNA sequence coding for the mature NFAP replaced the part of the *paf*-gene that codes for the mature PAF in the pSK275*paf* vector. The *nfap* cDNA was fused to the *paf* pre-pro sequence (pSK275*nfap* ^*paf_signal*^) and NFAP was produced in *P. chrysogenum* under the regulation of the strong *paf*-promoter (NFAP[Pc]) (Fig. 1a, c; Additional file 1: Fig. S2).

The production of PAF[Pc], PAF^F31N^, PAF^Y48Q^ and NFAP[Pc] required the use of the *P. chrysogenum* ∆*paf* mutant as cell factory, where the *paf* coding sequence was deleted by replacement with the nourseothricin (*nat1*) resistance gene [29].

To test the applicability of the expression cassette in other *Penicillium* species and with different transformation techniques (i.e., *Agrobacterium tumefaciens* mediated transformation, ATMT), we constructed an *A. tumefaciens* transformation vector with the expression cassette for *paf* gene insertion into the *P. digitatum* PHI26 genome (Fig. 1b). This experiment was intended to produce the *P. chrysogenum* PAF in *P. digitatum* (PAF[Pd]).

After single spore selection of positive *P. chrysogenum* and *P. digitatum* transformants, candidate clones were tested for highest protein production in time course experiments using small-scale fermentation. One clone each with the highest production of the desired recombinant protein was selected for further characterization. The respective producer strains were named *P. chrysogenum paf*, *P. chrysogenum paf* ^F31N^, *P. chrysogenum paf* ^Y48Q^, *P. chrysogenum nfap* and *P. digitatum paf.*

In Southern blotting experiments the random integration of the transforming DNA into the fungal genomes of the selected candidate clones was proved by using a 1.3 kb DIG-labelled PCR probe partially spanning the *paf* gene and the *paf* promoter (Additional file 1: Fig. S3). In all *P. chrysogenum* transformants the *nat1* resistant gene was still present that originated from the deletion of the *paf* gene in the *P. chrysogenum* Δ*paf* strain [29] (Additional file 1: Fig. S3). This correlated well with a nourseothricin-resistant phenotype of the respective transformants, in addition to the resistance for pyrithiamine acquired by the uptake of the pSK275*paf*-based plasmids. In *P. chrysogenum*, the random plasmid integration resulted in a hybridizing fragment of 2.6 kb in length (*P. chrysogenum nfap* strain digested with *Nhe*I/*Xho*I) or of 2.7 kb in length (*P. chrysogenum paf*/*paf* mutants digested with *Nde*I) and additional fragments of larger or smaller sizes, depending on the integration locus. The additional signals proved that single or multiple-copy random plasmid integrations in the fungal genomes had occurred (Additional file 1: Fig. S3). The analysis of genomic DNA of *P. digitatum paf* strain revealed the presence of at least three copies of the *paf* expression cassette. No signals were detected in the *P. digitatum* PHI26 recipient strain that lacks the *paf* gene (Additional file 1: Fig. S3).

The selected clones were cultivated under larger culture conditions and, after clearing the culture broth from insoluble matter, the proteins in the supernatant were purified by cation-exchange chromatography. Optimal protein production was achieved at 72–96 h of cultivation for PAF, PAF mutants and NFAP in *P. chrysogenum*, whereas longer cultivation times of 7–11 days were applied for high yield expression of PAF[Pd] in *P. digitatum paf*, due to a lower proliferation rate of this strain in minimal medium. The protein amounts varied between a few milligrams per litre up to approximately 80 mg/L (Table 1).

**Table 1:** Properties of recombinant proteins produced with the *P. chrysogenum*-based expression system

Proteins	Molecular mass [Da]^a^	Protein yield [mg/L]	Cultivation time [h]
PAF[Pc]	6244	80	72
PAF^F31N^	6211	35	72
PAF^Y48Q^	6209	60	72
NFAP[Pc]	6620	3	96
PAF[Pd]	6244	83	11 days

^a^Calculated theoretical molecular masses (ExPASy ProtParam tool)

**Fig. 2:**
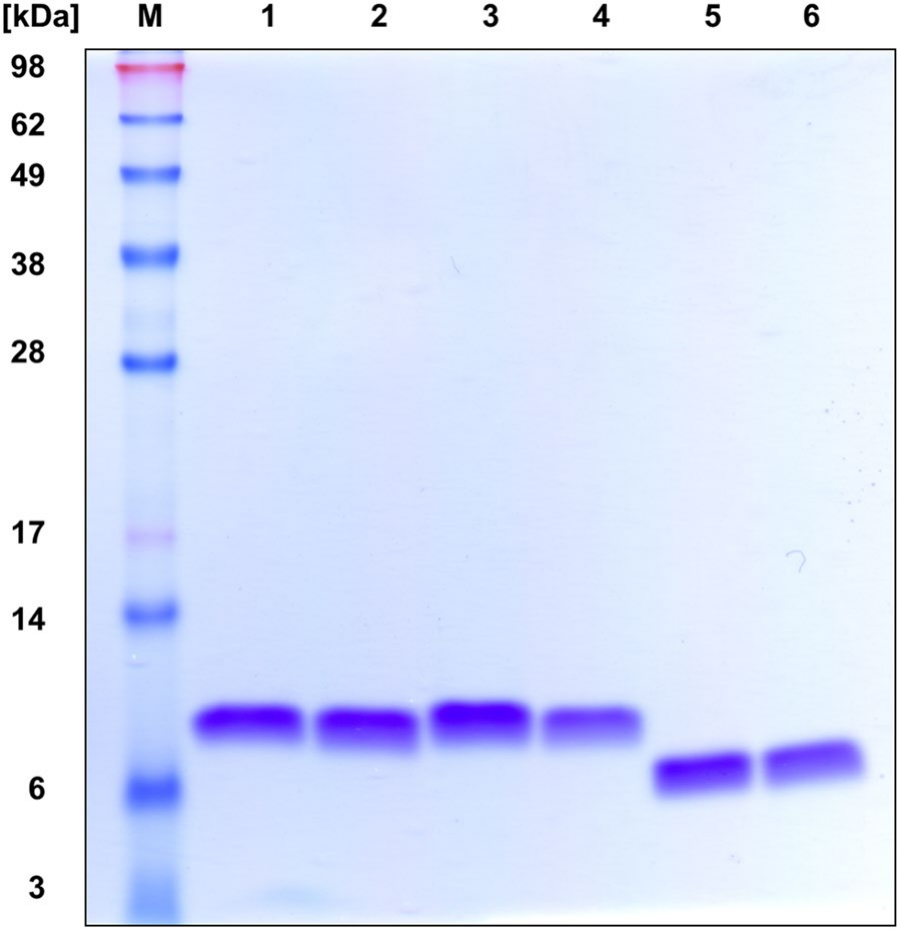
Analysis of purified recombinant proteins with 18% (w/v) tris–glycine SDS-PAGE. One microgram of the protein samples was loaded per lane and visualized by Coomassie blue staining. *M* pre-stained protein size-standard (SeeBlue Plus2, ThermoFischer Scientific, Waltham, MA, USA), *1* PAF[Pc], *2* PAF^F31N^, *3* PAF^Y48Q^, *4* PAF[Pd], *5* NFAP, *6* NFAP[Pc]

**Fig. 3:**
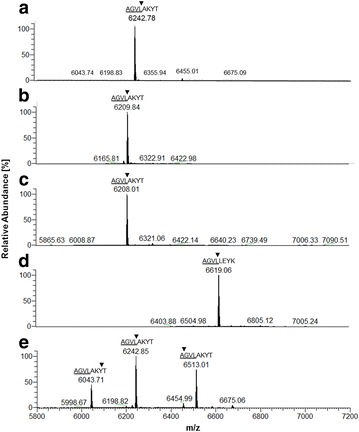
ESI-MS data showing the isotopic average molecular mass (m/z) of **a** PAF[Pc], **b** PAF^F31N^, **c** PAF^Y48Q^, **d** NFAP[Pc] and **e** PAF[Pd]. The last four amino acid residues of the pro sequence (*underlined*), the first four of the predicted mature proteins, and the experimentally determined N-terminal cleavage sites (*arrowheads*) are shown on each major MS peak

The purity of the protein preparations was verified with SDS-PAGE (Fig. 2) and ESI–MS analysis (Fig. 3). In the SDS-PAGE one single band was visible for each purified protein sample corresponding to a molecular weight of approximately 6 kDa. Interestingly, the slightly larger NFAP protein (6.6 kDa) showed faster migration than the other proteins (6.2 kDa). A slight deviation from the expected migration behaviour is a phenomenon often observed with small, cysteine-rich proteins [30, 31].

### Mass spectrometry

The identity and purity of all produced proteins and the correct amino acid conversions in case of PAF mutants were determined by ESI–MS. The average molecular (mol.) masses shown in Fig. 3 fit perfectly to the calculated theoretical mol. masses shown in Table 1 where the presence of three disulphide bridges in all proteins was taken into account. The PAF[Pc] mol. mass (6242.8 Da, Fig. 3a) was identical with that of PAF produced in *P. chrysogenum* Q176 [13]. The mol. mass of PAF^F31N^ (6209.8 Da) and PAF^Y48Q^ (6208.0 Da) proved the correct exchange of the Phe and Tyr residues for an Asn and Gln residue, respectively (Fig. 3b, c). The mol. mass of NFAP[Pc] (6619.1 Da, Fig. 3d) corresponded to the calculated 6620 Da (Table 1). The PAF[Pd] MS revealed one main signal of 6242.9 Da and two additional signals that correspond to PAF with variable N-terminus: one lacking the first two amino acids Ala-Lys (6043.7 Da) and one comprising three additional amino acids Gly-Val-Leu (6513.01 Da) at the N-terminus (Fig. 3e). However, this PAF[Pd] preparation produced one single peak during chromatographic purification (Additional file 1: Fig. S4) and a single band in SDS-PAGE analysis (Fig. 2). All MS data indicated that the six cysteine residues present in all proteins were oxidized and three intra-molecular disulphide bonds were formed during intracellular protein processing. Furthermore, the data excluded any further post-translational protein modifications, except for the cleavage of the pre-pro sequence.

### Antifungal activity assay

The antifungal activity of all recombinant proteins was tested on the PAF- and NFAP-sensitive model organism *A. niger* and the minimal inhibitory concentration (MIC) was determined (Table 2; Additional file 1: Fig. S5). Susceptibility data demonstrated that PAF[Pc] and PAF[Pd] showed the same antifungal activity as the wild-type PAF from *P. chrysogenum* Q176 (MIC 1.2 μg/mL; Additional file 1: Fig. S5). The recombinant NFAP[Pc] exhibited identical activity as the wild-type NFAP from *N. fischeri* (MIC 1.2 μg/mL; Additional file 1: Fig. S5). In contrast, PAF^F31N^ and PAF^Y48Q^ dramatically lost antifungal activity: for PAF^F31N^ the MIC was 800 μg/mL, whereas for PAF^Y48Q^ no MIC could be determined at the concentrations tested (Table 2; Additional file 1: Fig. S5).

**Table 2:** The MIC of the recombinant antifungal proteins on *A. niger*

Proteins	MIC [µg/mL]
PAF[Pc]	1.2
PAF[Pd]	1.2
PAF^F31N^	800
PAF^Y48Q^	>800
NFAP[Pc]	1.2

### ECD spectroscopy

**Fig. 4:**
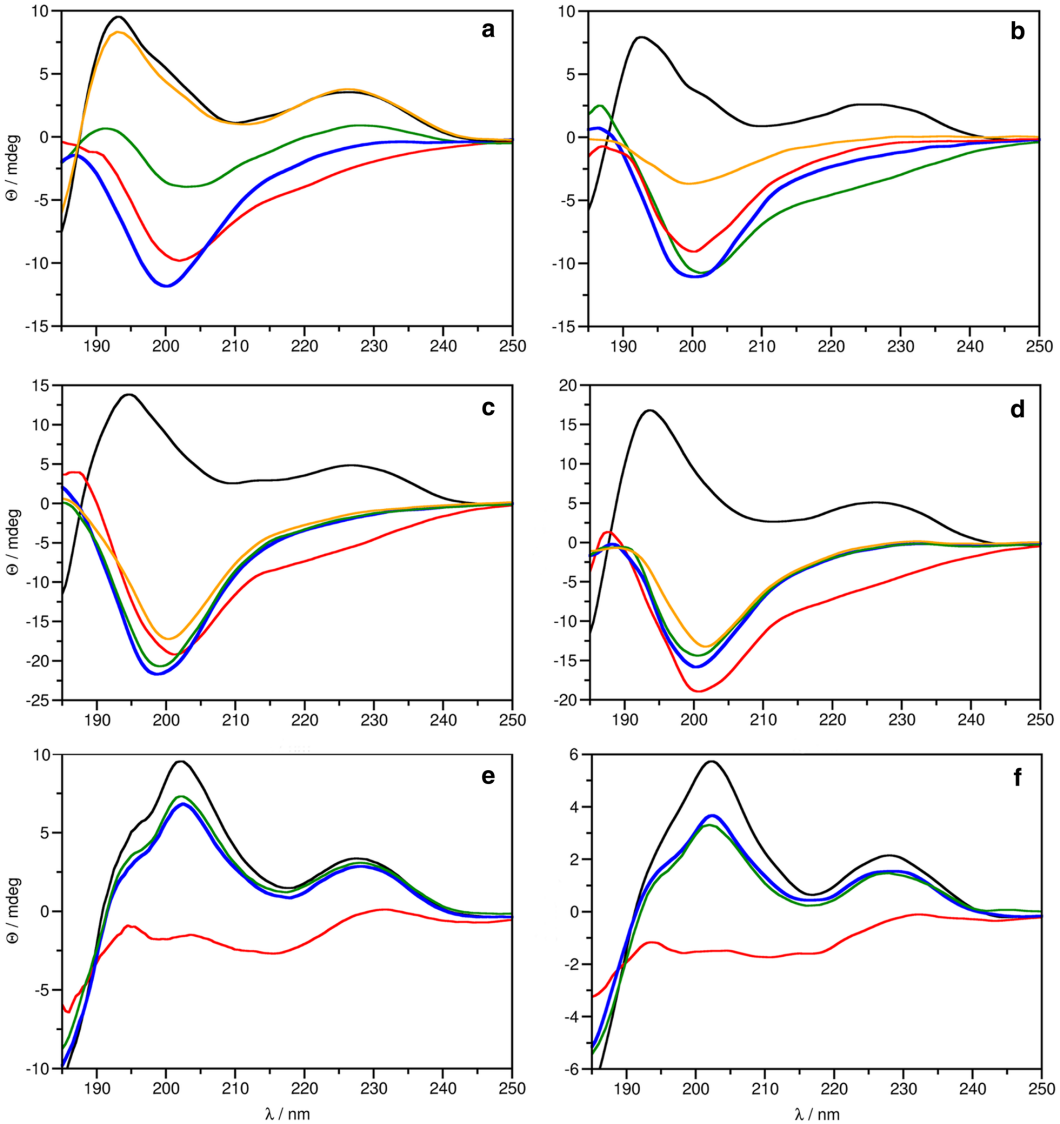
ECD spectra of** a** PAF[Pc],** b** PAF[Pd],** c** PAF^F31N^,** d** PAF^Y48Q^,** e** NFAP and** f** NFAP[Pc], recorded at 25 °C (*black*), 95 °C (*red*), and at 25 °C immediately (*blue*), 72 h (*green*) and 4 weeks (*orange*, only in** a**–**d**) after annealing

**Fig. 5:**
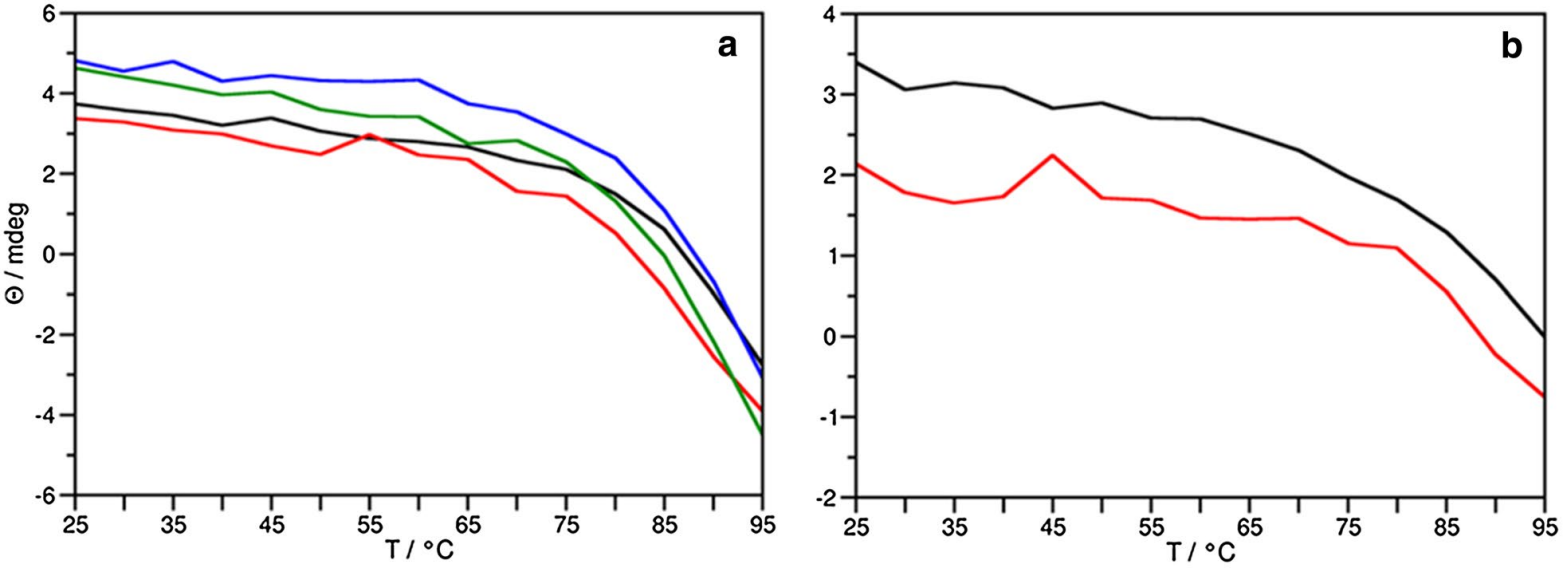
Thermal unfolding of** a** PAF[Pc] (*black*), PAF[Pd] (*red*), PAF^F31N^ (*green*), PAF^Y48Q^ (*blue*) and** b** NFAP (*black*) and NFAP[Pc] (*red*) followed by ECD spectroscopy at 229 nm

ECD spectroscopy is a sensitive tool for the determination of the secondary structure of proteins. Low sample volume (0.1–1 mL) and concentration (µM range) requirements make this method a sensible choice for the rapid determination of protein conformation in order to verify if an expressed, purified protein is correctly folded, or how different mutations may affect its structure and thermal stability [32]. The ECD spectrum of PAF[Pc] at 25 °C was highly similar to that reported previously for this protein [26] and other disulphide bridged, β-structured proteins [33] (Fig. 4a). The spectrum had two maxima at 195 and 229 nm, a low intensity minimum centred at 210 nm and a weak shoulder at around 200 nm. The maximum at 229 nm was mainly attributed to the presence of disulphide bridges while the low intensity minimum at 210 nm indicated β-pleated conformation. The maximum centred at around 195 nm reflected contributions from both β-pleated conformation and the spectral transitions of disulphide bridges. The spectrum of PAF[Pd] at 25 °C (Fig. 4b) was almost identical to that of PAF[Pc]. The spectra of PAF^F31N^ and PAF^Y48Q^ at this temperature (Fig. 4c, d, respectively) were similar too, differing only in the shoulder at 200 nm, which was missing or less pronounced in the spectra of these PAF mutants. In general, ECD spectra measured at 25 °C indicated that the structure of PAF[Pd] and the two PAF mutants are highly similar to the native fold of PAF. Spectra measured at 95 °C reflected the loss of ordered secondary structure in case of all PAF proteins ([Pc], [Pd] and mutants). After heat treatment and cooling back to 25 °C, gradual structural reorganization of PAF[Pc] and PAF[Pd] took place (Fig. 4a, b). However, this reorganization was very slow and in the case of PAF[Pd] refolding was incomplete even after four weeks (Fig. 4a, b; yellow line). This observation may be attributed to the variation of the N-terminus in PAF[Pd]. In contrast to PAF[Pc] and PAF[Pd], thermal unfolding of PAF mutants was irreversible, even after four weeks (Fig. 4c, d). The unfolding curves of PAF proteins (Fig. 5a) reflected their high thermal stability, which was not affected by the amino acid exchanges in the PAF mutants or when PAF was expressed in *P. digitatum*. These curves also indicated that unfolding was not complete in the studied temperature range that reached 95 °C as the curves did not present the usual sigmoidal shape. Nevertheless, the loss of secondary structure was apparent on the spectra measured at high temperatures (Fig. 4). The data of the unfolding experiment did not allow the fitting of a sigmoidal function of which inflexion point would have defined the melting temperature (T_m_) of the protein structure. Hence T_m_ of the PAF proteins could only be estimated to be around 85 °C.

The ECD spectrum of the native NFAP (Fig. 4e) and the recombinant NFAP[Pc] (Fig. 4f) measured at 25 °C displayed similar features as the PAF spectra presented above, which suggests that this closely related protein has similar structural features as PAF[Pc]. The main difference between the spectra of NFAP and PAF[Pc] was the intensity ratio of the two components (195 and 200 nm) of the large positive maximum to be the opposite from that observed for PAF[Pc]. This might be due to a slightly different disulphide bridge pattern and/or different relative contribution of β-sheets to the overall structure of the protein. The spectrum of NFAP measured at 95 °C indicated only partial unfolding of the protein. The band at 229 nm reflecting the presence of disulphide bridges remained present opposed to PAF[Pc] where the dominance of unordered structures was observed at this temperature. Moreover, after cooling back to 25 °C the native fold of NFAP was restored completely and immediately. The heterologous expressed NFAP[Pc] adopted the same three-dimensional structure as NFAP and behaved in the same manner when heated and then cooled (Fig. 4f). The thermal stability of NFAP[Pc] appeared to be similar to that of NFAP, although the T_m_ was again roughly estimated (>85 °C) (Fig. 5b).

### NMR analysis

**Fig. 6:**
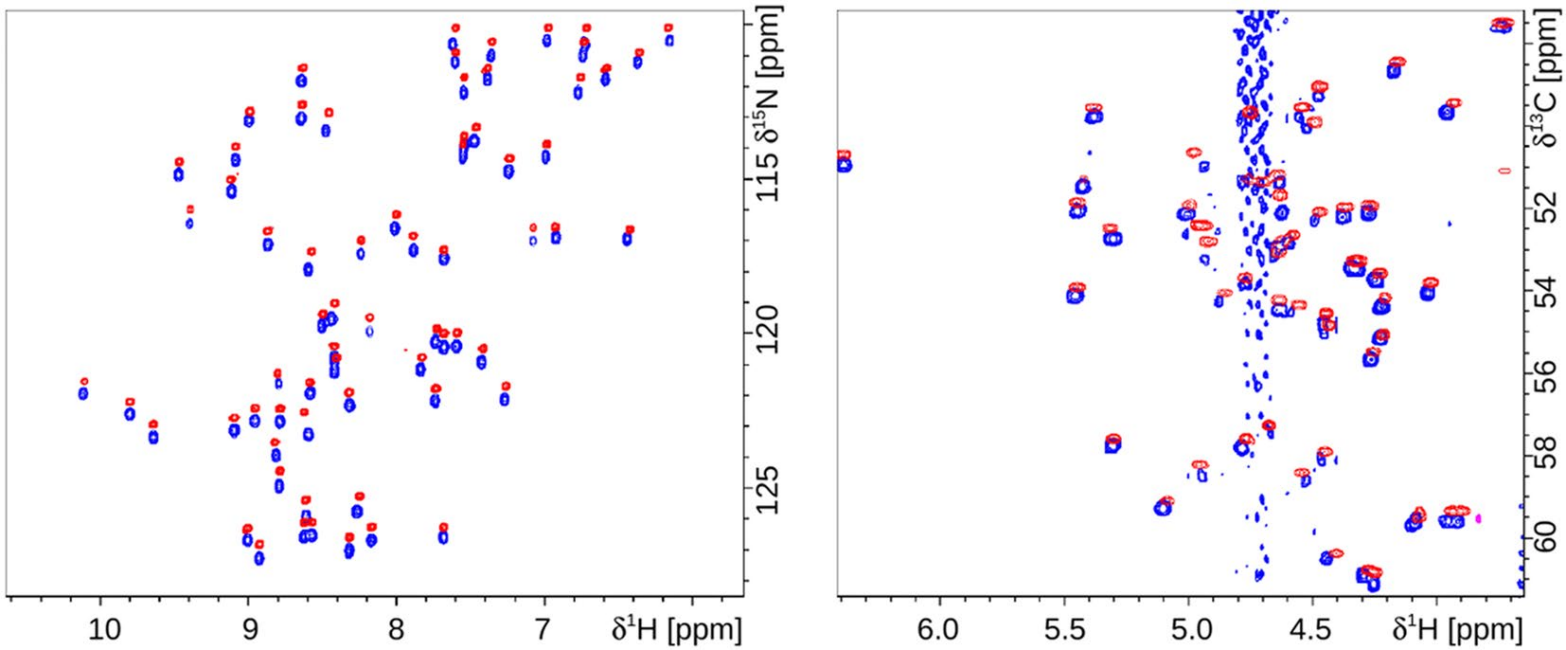
Overlaid view of ^15^N–^1^H HSQC (*left*) and Cα-Hα region of ^13^C-^1^H HSQC (*right*) of ^15^N-labelled PAF produced in the wild-type *P. chrysogenum* Q176 (*blue*) and ^15^N/^13^C-PAF[Pc] (*red*). Chemical shift scale was intentionally shifted in the F1 dimension for clarity

To provide a proof of principle that the *P. chrysogenum* expression system is suitable for the production of uniformly isotope-labelled and pure proteins for NMR analyses, PAF[Pc] was ^15^N-labelled or ^15^N-and^13^C-labelled (^15^N/^13^C-PAF[Pc]), purified and analysed by heteronuclear NMR experiments. These experiments provide direct information about the chemical environment of almost all atoms within a protein at atomic resolution (all hydrogens which are directly attached to a nitrogen or a carbon atom are generally resolved in the heteronuclear dimension). The lack of NMR signals from unlabelled PAF[Pc] in single or double labelled samples proved that the incorporation of stable isotopes was close to 100%. The comparison of the ^15^N-^1^H and ^13^C-^1^H heteronuclear single quantum coherence (HSQC) spectra of the ^15^N/^13^C-PAF[Pc] sample to the previously recorded NMR data of ^15^N-labelled or ^15^N/^13^C-labelled PAF generated in *P. chrysogenum* wild-type Q176 demonstrated the absolute spectral identity of these protein samples [13, 26] (Fig. 6).

**Fig. 7:**
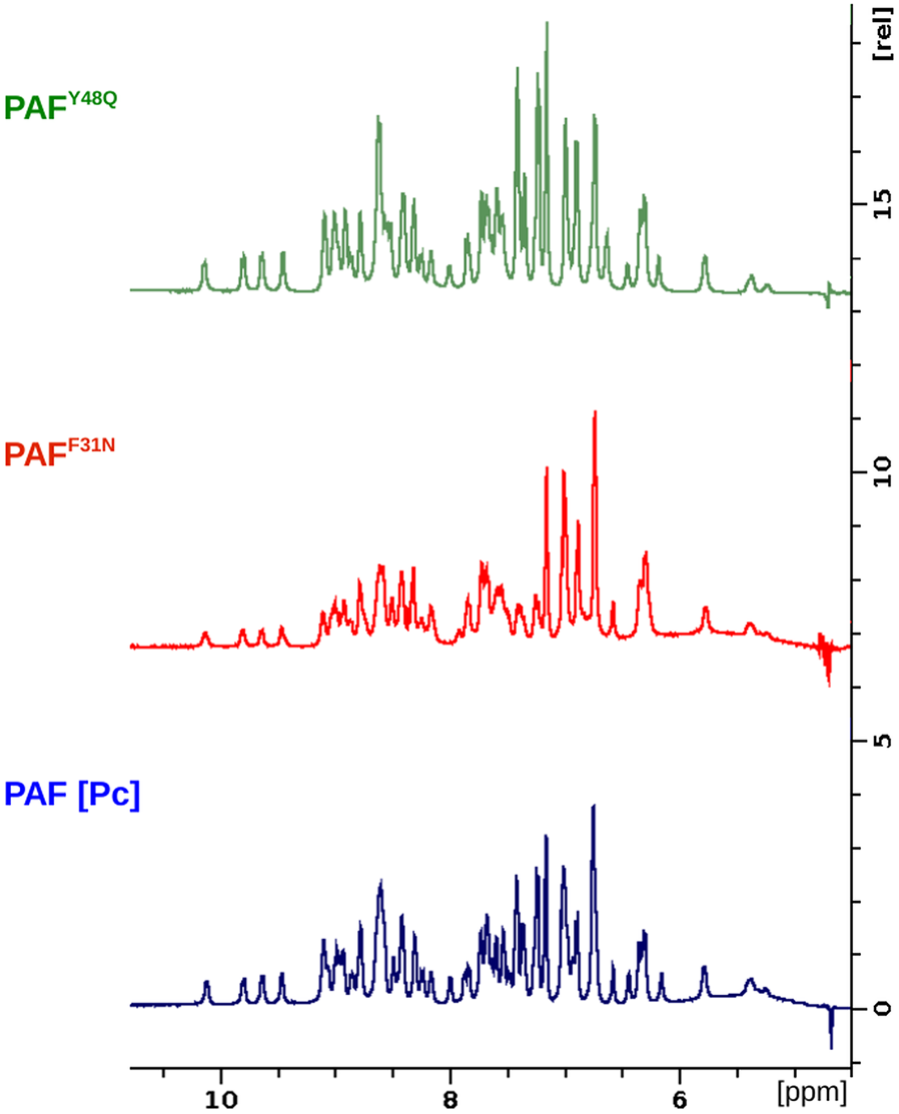
Amide and aromatic signal region of the ^1^H-NMR spectra of PAF[Pc], PAF^F31N^ and PAF^Y48Q^. Sharp signals and dispersion indicate the folded state of the analysed proteins

^1^H homonuclear spectra of PAF[Pc], PAF^F31N^, and PAF^Y48Q^ were acquired using solvent water suppression scheme (Fig. 7). The excellent dispersion of the amide signals in the ^1^H-NMR spectra proved the correct processing and folding of PAF[Pc] and the PAF mutants. These results were consistent with the data generated by ECD spectroscopy (Fig. 6). Moreover, all the samples were found to be pure by NMR standards: signals of impurities or minor forms of the products were not observed or were negligible for the given detection limit of the 500 MHz NMR spectrometer.

## Discussion

There is a strong interest in investigating the potential of natural molecules for the development of new antifungal strategies. Filamentous ascomycetes are valuable and promising sources for various APs most of which await identification and further characterization [7]. This requires the generation of considerable protein amounts for analyses. The expression of APs however, varies significantly under laboratory conditions and might be low or repressed in some cases.

To overcome these restrictions, we introduced in this study a new expression system for the recombinant production of small cysteine-rich APs that are generally difficult to obtain in a soluble and correctly folded conformation when expressed in heterologous systems [34]. Correct disulphide bond pattern and protein folding, however, are essential for full antifungal activity [13]. Furthermore, we could show that the cultivation of two different *Penicillium* spp. in defined minimal medium for protein expression allowed single-step purification and provided high yields of proteins without unwanted carbohydrate impurities or posttranslational protein modifications for structural analyses using ECD spectroscopy and NMR techniques with ^15^N- or ^15^N/^13^C-labelled proteins.

Optimal AP production in *P. chrysogenum* under the control of the *paf* promoter required the use of the ∆*paf* mutant to avoid co-expression of the wild-type PAF. In contrast, the PAF-related AP of *P. digitatum* (AfpB) could not be detected so far even under constitutive expression conditions of the respective *afpB* gene [7] and therefore, the wild-type *P. digitatum* PHI26 was applied as recipient strain in this study. Here we could show that the promoter, the pre-pro and terminator sequences of the *P. chrysogenum paf* gene work efficiently also in a heterologous system, e.g. to produce PAF in *P. digitatum* with high yields. The additional MS signals detected in PAF[Pd] may indicate potential problems of *P. digitatum* proteases with the recognition of the pro sequence during protein maturation. However, the processing of the signal sequence and the disulphide bond formation seems to work properly as high yields of correctly folded and bioactive PAF[Pd] were recovered from the supernatant. The biological function of the pro sequence in APs is less understood, but a chaperone-like role for proper protein folding is suggested [1]. Naturally occurring variations of the N-terminus have been observed in related, non-recombinant APs, such as the *A. giganteus* AFP [35]. However, our data demonstrate that subtle differences in the processing of the pro sequence do not affect the antifungal activity of PAF[Pd].

The *N. fischeri* AP NFAP slightly differs from PAF in predicted structure, antifungal spectrum and mechanistic function [3–5]. Despite the knowledge of the transcriptional regulation elements, the bulk production of pure NFAP for structural investigations has not been achieved yet in the native producer *N. fischeri* NRRL181 where the average NFAP yield was not more than 1 mg/L [4]. It was previously proved that *P. pastoris* KM71H produces high amounts (average yield 6 mg/L) of folded and active recombinant NFAP [4], but the carbohydrate impurities as a consequence of the secretion system severely disturbed the structural analyses. In this study, we adopted the *P. chrysogenum* ∆*paf* expression system to produce this protein. Two different pSK275*paf*-based expression vectors were constructed for the production of NFAP in *P. chrysogenum* ∆*paf*. The attempt to put the NFAP encoding cDNA with the *nfap* specific pre-pro sequence (pSK275*nfap*) under the control of the strong *paf* promoter did not succeed in raising the NFAP protein yield (unpublished data). Instead, the average NFAP[Pc] yield of the pSK257*nfap* ^*paf_signal*^ transformant was approximately 3 mg/L, three-fold higher than in the natural producer *N. fischeri* NRRL181. This amount was sufficient for the structural and functional experiments in this study and isotope-labelling of NFAP[Pc] is in progress to resolve the three dimensional structure by NMR in the near future.

Furthermore, our data indicate that the gene copy number and the protein yield did not directly correlate. However, the NFAP[Pc] yield did not reach that of the other recombinant proteins. This could be a consequence of the genomic integration site and/or the high copy-number of the plasmid pSK257*nfap* ^*paf_signal*^ in the *Penicillium* genome. High gene dosage was shown to possibly hamper the transcription or translation machinery of microbial cell factories [36, 37]. However, protein-specific determinants, such as the signal sequence, may also modulate heterologous protein production, processing and secretion in the *Penicillium* sp. cell factories [38]. To elucidate the molecular background that regulates these processes further investigations are currently in progress.

**Fig. 8:**
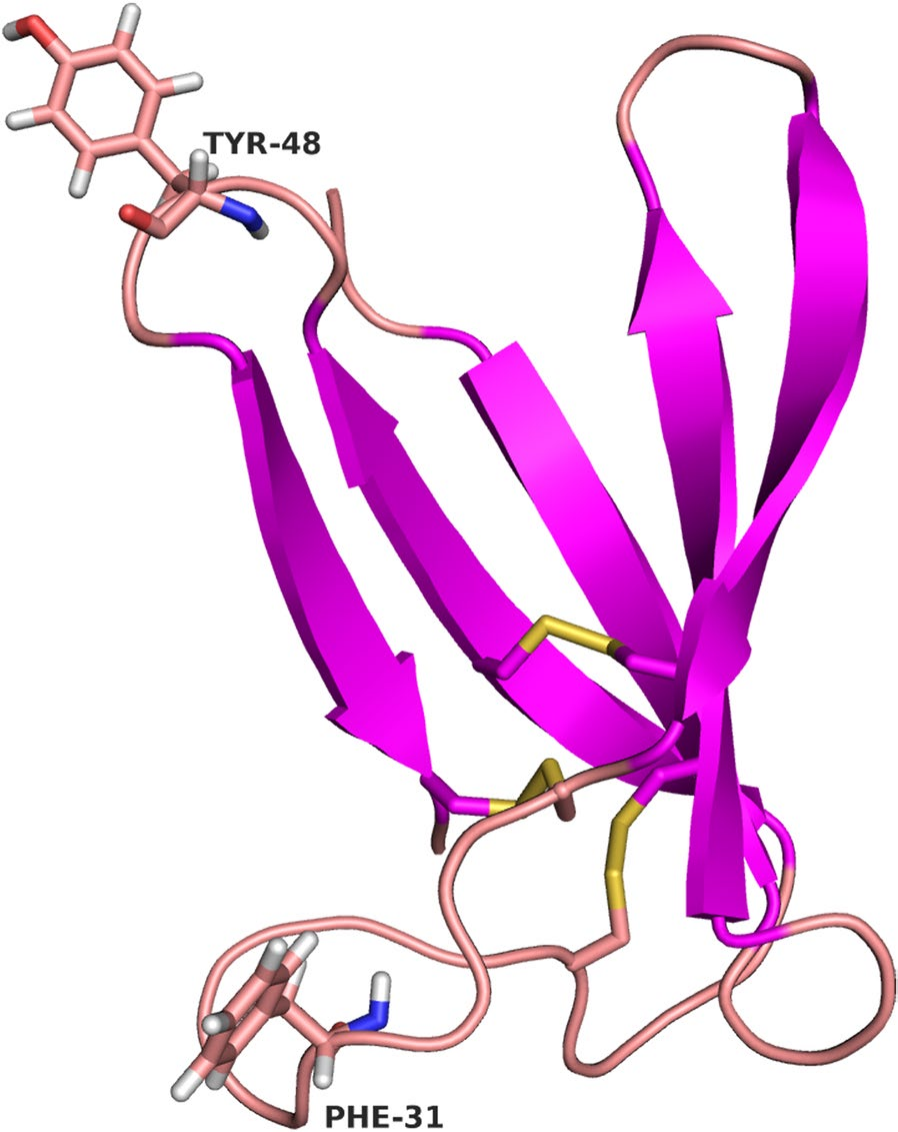
Cartoon representation of the PAF solution structure. *Purple arrows* show β-sheets, lines in *light red* indicate the loops, *yellow lines* highlight the disulphide bridges between the corresponding cysteines. The residues Phe31 and Tyr48 are indicated

Since we are interested to unravel the structure–function relation of PAF for a better understanding of its antifungal mode of action, we took advantage of the *P. chrysogenum* ∆*paf* cell factory to express two PAF mutants PAF^F31N^ and PAF^Y48Q^ with exchanged hydrophobic residues (Phe, Tyr) at position 31 and 48, respectively (Fig. 8).

The Phe31 participates in the most extended hydrophobic region of PAF with low primary sequence similarity to related APs [7, 13, 38], whereas Tyr48 represents a highly conserved residue that forms, together with two other tyrosines (Tyr3 and Tyr50), a well-defined aromatic region in PAF [13]. Both amino acids reside in loop regions of PAF, Phe31 in loop 3 and Tyr48 in loop 4, and are surface and solvent exposed. These loop regions show increased mobility and structural heterogeneity, which points towards a role as possible interaction sites with fungal target molecules [13, 26]. The replacement of these two residues by the neutral residues Asn and Gln resulted in a dramatic loss of activity and refolding capability (Fig. 4), underlining the important role of the postulated hydrophobic and aromatic patch in PAF for full antifungal function. Importantly, the data acquired by ECD spectroscopy and NMR analyses proved that the loss of activity could not be assigned to unfolding or misfolding of the protein mutants. The presence of β-sheets characteristic for the secondary structures of PAF[Pc] and PAF mutants PAF^Y48Q^ and PAF^F31N^ was proven by the well dispersed ^1^H and/or ^1^H/^15^N HSQC NMR spectra, represented by the fingerprint amide spectral regions (Figs. 6, 7). The *abcabc* type disulphide pattern was unambiguously verified for PAF (pdb code: 2mhv, RCSB:10362) [25, 26] and corresponded to the pattern suggested for the *A. giganteus* AFP (*abcdabcd*) [38]. Cysteines that are oxidized and form intramolecular disulphide bonds elicit cysteine β ^13^CH_2_ signals that are well above 35 ppm. In case of PAF[Pc], the signals reached 37–39 ppm (NMR data of PAF can be found at BMRB code: 19657). Therefore, the *abcabc* pattern is highly probable for PAF[Pc] and the PAF mutants. If the disulphide bonds were intentionally broken by heat and/or reducing agents, the scrambled protein aggregates would result in much less dispersed NMR signals typical for unstructured proteins. Accordingly, the disappearance of the intense positive band at 229 nm from ECD spectra recorded at elevated temperatures could be attributed to either the disruption or conformational change of disulphide bridges during this measurement [39]. It has been reported that UV excitation of aromatic residues may result in electron or H ejection, which can reduce nearby disulphide groups [40]. Both the PAF and NFAP sequence contains aromatic amino acid residues adjacent to cysteines. Such UV light-induced reduction may occur at elevated rate at high temperatures (95 °C). Disruption of disulphide bridges and formation of possible new disulphide bonds in random oligomers at the experimental conditions applied could explain the apparent differences between the ECD spectra regarding the unfolding of PAF, NFAP and especially of PAF mutants.

## Conclusion

We developed an expression cassette for homologous and heterologous gene expression under the strong *paf*-promoter for its application in *P. chrysogenum* and *P. digitatum* using different transformation techniques. This system was used to successfully increase the yield of correctly folded, small, cysteine-rich and cationic APs. Single-step purification from the *Penicillium* spp. culture broth of defined minimal nutrient composition ensured high protein quality and uniform isotope-labelling suitable for structural analyses employing NMR technology and ECD spectroscopy. NMR-based techniques provide information about the structure and dynamics of macromolecules. Since even the most recent NMR spectrometers are relatively insensitive, e.g. compared to MS, a protein concentration of 0.1–1 mM in a 250–500 µL sample volume is generally needed. Efficient protein production is especially important when an expensive ^13^C-labelled carbon source is utilized. Therefore, any improvement in protein yield and purity makes the research more cost-effective and supports approaches to investigate protein function, applying for example alanine scan methodology [41, 42]. The availability of restriction enzyme free cloning strategies, such as the seamless cloning technique [43], allows a broad application of our expression cassette using the strong *paf* promoter/terminator. *Penicillium* sp. represents an ideal microbial cell factory for the expression of a multitude of genes, originating also from non-fungal species [20], as protein processing and maturation is similar to higher eukaryotes [44, 45]. Taking into account the recent approaches for “humanizing” the glycosylation profile (as one of the most common post-translational modifications) in filamentous fungi [27], the fungal expression system presented in this study could be further refined in the future for the expression of human therapeutic proteins. This system is exceptionally promising for studying structure–function relations supporting the rational design of biotechnological interesting proteins and peptides.

## Methods

### Strains and growth conditions

Plasmids and vectors were propagated in *E. coli* DH5α, grown in LB medium [1% tryptone, 0.5% yeast extract, 1% NaCl (w/v)], supplemented with 100 μg/mL ampicillin. Fungal strains used in this study are listed in Table 3. For transformation*, P. chrysogenum ∆paf* [29] shaking cultures were grown in complete medium (CM; Additional file 1: Table S1) for 36 h at 25 °C. Conidia were harvested with spore buffer (0.9% NaCl (w/v), 0.01% Tween 80 (v/v)). For protein production, 2 × 10^8^ conidia of *P. chrysogenum* or *P. digitatum* were inoculated per 200 mL PcMM or 450 mL PdMM, respectively (Additional file 1: Table S1) and cultivated at 25 °C under continuous shaking (200 rpm). Isotopic ^15^N- and/or ^15^N/^13^C-labelling of recombinant proteins for NMR analysis was performed by replacing the nitrogen/carbon source by 0.3% Na^15^NO_3_ and 1% ^13^C-glucose, respectively (Euriso-Top, Saarbrücken, Germany) in PcMM. As a PAF- and NFAP-sensitive test organism *A. niger* was cultivated in 20-fold diluted potato dextrose broth (0.05 × PDB, Sigma-Aldrich, St Louis, MO, USA) for growth inhibition assays. *A. niger* conidia were harvested from cultures grown at 37 °C on CM agar (Additional file 1: Table S1). Single use working solutions of conidia were prepared by adding 10% glycerol (v/v) to conidia suspensions, freezing them in liquid nitrogen and storing at –80 °C.

**Table 3:** Fungal strains used in this study

Strain	Genotype	Reference
*A. niger*	wild-type	CBS 120.49
*P. chrysogenum* Q176	wild-type	ATCC 10002
*P. chrysogenum ∆paf*	*∆paf*:*nat1*	[29]
*P. chrysogenum paf*	*∆paf*:*nat1, paf*, *ptrA* ^+^	This study
*P. chrysogenum paf* ^F31N^	*∆paf*:*nat1, paf* ^F31N^, *ptrA* ^+^	This study
*P. chrysogenum paf* ^Y48Q^	*∆paf*:*nat1, paf* ^Y48Q^, *ptrA* ^+^	This study
*P. chrysogenum nfap*	*∆paf*:*nat1, nfap* ^*paf_signal*^, *ptrA* ^+^	This study
*P. digitatum* PHI26	wild-type	CECT 20796
*P. digitatum paf*	*paf, hph* ^+^	This study

### Vector constructions

All PCR reactions were performed with Q5 High Fidelity DNA Polymerase (New England Biolabs, Ipswich, MA, USA). From the plasmid pSK275 [28], pSK275*paf* was created by inserting the *paf* gene (420 bp) and approximately 1280 bp of the 5′-UTR and 370 bp of the 3′-UTR (Fig. 1; Additional file 1: Fig. S1). To generate PAF protein mutants, site-directed mutagenesis was applied to pSK275*paf*, using specific mutation primers that included mismatch nucleotides encoding for the amino acid to be exchanged (Additional file 1: Table S2). First, two PCR reactions were carried out with the primer pairs M13 and the respective forward (fw) mutation primer or opaf10 and the respective reverse (rev) mutation primer. In the next step, the generated fragments were combined in a PCR with the primers T7var and opaf9. The received mutated *paf* gene was cloned into pSK275*paf* at its *Not*I/*Nru*I restriction sites. Thus the plasmids for protein expression were named pSK257*paf* ^F31N^ and pSK257*paf* ^Y48Q^.

The expression cassette for NFAP production was constructed by preparing a PCR fragment from plasmid pBSK(-)*nfap* [4] containing the cDNA encoding the mature NFAP. This was fused to the *paf* pre-pro sequence and flanked by parts of the *paf* 5′-UTR and 3′-UTR. The DNA fragment was cloned into the *Bsp*MI and *Not*I digested pSK275*paf* vector, exchanging the *paf*-coding sequence (pSK275*nfap* ^*paf_signal*^). A detailed description of the cloning strategy is given in the Additional file 1: Fig. S2.

To generate the *P. digitatum paf* strain, the *paf* expression cassette was obtained from the pSK275*paf* vector by PCR amplification, using the primers OJM483 and OJM484 to introduce restriction sites *Xma*I and *Xba*I, respectively (Additional file 1: Table S2). This cassette was first subcloned into the pGEM^®^-T Easy vector system (Promega, Fitchburg, WI, USA), from where it was excised with *Xma*I and *Xba*I and inserted into the appropriately digested binary vector pBHt2 (pBHt2_PAF), containing the hygromycin (*hph*) resistance selection marker [46].

In all cases, the correct vector assembly and nucleotide sequences were verified with Sanger sequencing.

### Fungal transformation and confirmation of gene integration

For the production of PAF, PAF mutants and NFAP in *P. chrysogenum* the *paf*-deletion strain *P. chrysogenum* ∆*paf* [29] served as a recipient for the plasmids pSK257*paf*, pSK257*paf* ^F31N^, pSK257*paf* ^Y48Q^, pSK257*nfap* ^*paf_signal*^. *P. chrysogenum* ∆*paf* was grown as described above and transformation was carried out according to [47, 48], using 10 µg of *Not*I linearized plasmids per transformation. Transformants were single spored three times on PcMM agar plates supplemented with 0.3–0.6 µg/mL pyrithiamine hydrobromide (Sigma-Aldrich, St Louis, MO, USA) to obtain genetically homogeneous strains.

For the production of PAF protein in *P. digitatum*, the binary vector pBHt2_PAF was transformed into *A. tumefaciens* AGL-1, and used to transform the parental CECT 20796 strain by ATMT as previously described [49, 50] with modifications as indicated in [51].

To confirm gene integration Southern blotting was performed. A DIG-labelled probe (1.3 kb) was PCR amplified using the *paf*-specific oligonucleotides intron1paf (within the *paf*-coding region) and opaf13 (within the *paf* 5′-UTR). Genomic DNA was extracted according to [52] and restriction enzyme digested. A detailed description is given in Additional file 1.

### Protein expression and purification

PAF, PAF mutants and NFAP were purified from the supernatants of *P. chrysogenum* and *P. digitatum*. Shaking cultures of *P. chrysogenum* were first cleared from mycelia. The cell-free supernatant was ultra-filtered (Ultracell 30 kDa, Millipore, Billerica, MA, USA) and applied to a CM-Sepharose (Fast Flow, GE Healthcare Life Sciences, Little Chalfont, UK) column, equilibrated in phosphate buffer (10 mM NaPO_4_, 25 mM NaCl, 0.15 mM EDTA, pH 6.6). The *P. digitatum* cell-free supernatant was dialyzed (2 K MWCO, Sigma-Aldrich, St Louis, MO, USA) against the phosphate buffer before applying to an AKTA Purifier system equipped with a 6 mL RESOURCE™ S column (GE Healthcare Life Sciences, Little Chalfont, UK), equilibrated in phosphate buffer. In all cases, the proteins were eluted applying 0.1-0.5 M NaCl. The protein containing fractions were pooled and dialyzed (3.5 K MWCO, ThermoFisher Scientific, Waltham, MA, USA) against ultra-pure ddH_2_O and filter sterilized (0.22 µm, Millex-GV, PVDF, Millipore, Billerica, MA, USA). Protein concentrations were determined spectrophotometrically (A_280_) considering the respective molar extinction coefficients and the purity was checked by SDS-PAGE using Coomassie blue staining.

### Mass spectrometry

The mass of the purified proteins was determined by ESI–MS on a CESI 8000 (AB Sciex, Framingham, MA, USA) coupled to a Q Exactive (ThermoFisher Scientific, Waltham, MA, USA) at the Protein Micro-Analysis Facility (Medical University of Innsbruck). PAF protein samples were directly diluted in 100 mM acetic acid and analysed (180 nL/min flow rate). NFAP samples were ZipTip (EMD, Millipore, Billerica, MA, USA) enriched, dissolved in 100 mM acetic acid and analysed by capillary electrophoresis (CE)-ESI–MS (20 kV separation voltage, 10 psi pressure). Protein masses were determined by deconvolution using the integrated Xcalibur pXtract software (ThermoFisher Scientific, Waltham, MA, USA).

### ECD spectroscopy

ECD spectroscopic measurements were performed in the 195–260 nm wavelength range (far-UV) to determine the secondary structure and examine the structural stability of the recombinant proteins. Protein samples were dissolved in pure ddH_2_O at approximately 0.1 mg/mL concentration and measured in a 0.1 cm path-length quartz cuvette using a Jasco J-815 spectropolarimeter (JASCO, Tokyo, Japan) at a scan speed of 100 nm/s. In brief, first ECD spectrum of the sample was recorded at 25 °C, then the temperature was increased gradually up to 95 °C at a rate of 1 °C/min using a Peltier thermoelectronic controller (TE Technology, Traverse City, MI, USA) while ellipticity data was recorded as a function of temperature at three wavelengths, appointed by the extrema of the spectrum measured at 25 °C. The system was allowed to equilibrate for 1 min at each temperature point before measurements were taken. The resulting melting curves were used to estimate the T_m_ of the protein structures. Then, an ECD spectrum in the 195–260 nm wavelength range was recorded at 95 °C, the final temperature point of the unfolding experiments. The sample was left to cool and the full spectrum was measured again 1 min after the temperature reached 25 °C. Spectrum acquisition was repeated 72 h later and when the observed changes made it necessary. The presented spectra are accumulations of 10 scans, from which the similarly recorded spectrum of ddH_2_O was subtracted. Ellipticity data were given in mdeg units.

### NMR analysis

All NMR experiments were performed with a Bruker Avance II 500 spectrometer equipped with TXI z-gradient probe head (Bruker, Rheinstetten, Germany). Typically, 2-10 mg lyophilized protein was dissolved in 275 μL buffer (10 mM Na_2_HPO_4_/NaH_2_PO_4_, pH 6.0, 5% D_2_O (v/v), 0.04% NaN_3_ (w/v), 40 mM NaCl) and filled into a Shigemi NMR tube (Shigemi, Allison Park, PA, USA). ^1^H-NMR experiments were recorded with the watergate-5 sequence [53] for water suppression and 64 transients were collected in every case at 297 K. Sensitivity enhanced HSQC experiment was performed for ^1^H-^15^N and ^1^H-^13^C correlation [54] that allowed to obtain spectra even at natural abundance. In these heteronuclear experiments 1024 × 128 and 2048 × 350 points were acquired, respectively.

### Antifungal activity assay

For testing the antifungal activity of the recombinant produced proteins, *A. niger* was used as a sensitive test organism. Susceptibility tests were carried out in 96-well, flat-bottom microtitre plates (Nunclon Delta, Thermo Scientific, Waltham, MA, USA) as described before [55]. Briefly, 5 × 10^3^ conidia/mL were incubated in 0.05 × PDB with increasing concentrations of APs (0–800 µg/mL) in a total volume of 200 µL. The plates were incubated at 30 °C for 48 h and the growth was monitored microscopically using an inverted microscope (Leica DM IL Led, Leica Microsystems, Vienna, Austria) equipped with an AxioCam MR digital camera (Zeiss, Jena, Germany) for imaging. The images were processed with AxioVision software (Zeiss, Jena, Germany). The MIC was defined as the minimal protein concentration that inhibited fungal growth by ≥90%. Experiments were prepared in triplicates and performed at least three times.

### Statistical analysis

Statistical analysis was performed using Microsoft Excel 2010 (Microsoft Corp.).

## Additional files

**Additional file 1.** **Table S1.** Composition of media used in this study.** Table S2.** Oligonucleotides used in this study.** Figure S1.** Nucleotide sequence of plasmid pSK275*paf* containing the *paf* expression cassette.** Figure S2.** Cloning strategy of pSK275*nfap* ^*paf_signal*^.** Figure S3.** Southern blot analysis of genomic integration of the* P. chrysogenum* expression cassettes in the* Penicillium* spp.** Figure S4.** Chromatogram of the purification of PAF[Pd]. **Figure S5.** Microscopic images of growth inhibition assays with *A. niger* exposed to PAF, PAF[Pc], PAF^F31N^, PAF^Y48Q^, PAF[Pd], NFAP and NFAP[Pc].

## Abbreviations

AFP: *Aspergillus giganteus* antifungal protein
AP: antifungal protein
ATMT: *Agrobacterium tumefaciens* mediated transformation
CM: complete medium
DIG: digoxigenin
ECD: electronic circular dichroism
ESI-MS: electro-spray ionization mass spectrometry
HSQC: heteronuclear single quantum coherence
MIC: minimal inhibitory concentration
MS: mass spectrometry
MWCO: molecular weight cut-off
NFAP: *Neosartorya fischeri* strain NRRL181 antifungal protein
NFAP[Pc]: recombinant NFAP produced in *Penicillium chrysogenum*
NMR: nuclear magnetic resonance
PAF: *Penicillium chrysogenum* strain Q176 antifungal protein
PAF[Pc]: recombinant PAF produced in *Penicillium chrysogenum*
PAF[Pd]: recombinant PAF produced in *Penicillium digitatum*
PcMM: *Penicillium chrysogenum* minimal medium
PdMM: *Penicillium digitatum* minimal medium
SDS-PAGE: sodium dodecyl sulphate polyacrylamide gel electrophoresis
UTR: untranslated region
