# Supplementary material for: A Penicillium chrysogenum-based expression system for the production of small, cysteine-rich antifungal proteins for structural and functional analyses
Source: Microb Cell Fact. 2016 Nov 11;15:192. doi: 10.1186/s12934-016-0586-4 (PMC5106836; doi:10.1186/s12934-016-0586-4)
Supplement: Supplementary file 1 — Additional file 1. Table S1. Composition of media used in this study. Table S2. Oligonucleotides used in this study. Figure S1. Nucleotide sequence of plasmid pSK275paf containing the paf expression cassette. Figure S2. Cloning strategy of pSK275nfap paf_signal. Figure S3. Southern blot analysis of genomic integration of the P. chrysogenum expression cassettes in the Penicillium spp. Figure S4. Chromatogram of the purification of PAF[Pd]. Figure S5. Microscopic images of growth inhibition assays with A. niger exposed to PAF, PAF[Pc], PAFF31N, PAFY48Q, PAF[Pd], NFAP and NFAP[Pc]. [file 12934_2016_586_MOESM1_ESM.docx]

**A *Penicillium chrysogenum*-based expression system for the production of small, cysteine-rich antifungal proteins for structural and functional analyses**

**Christoph Sonderegger^1^, László Galgóczi^1^, Sandra Garrigues^2^, Ádám Fizil^3^, Attila Borics^4^, Paloma Manzanares^2^, Nikoletta Hegedüs^1§^, Anna Huber^1^, Jose F. Marcos^2^, Gyula Batta^3^, Florentine Marx^1*^**

**^1^**Division of Molecular Biology, Biocenter, Medical University of Innsbruck, Innrain 80-82, 6020 Innsbruck, Austria

^2^Biotechnology Department, Instituto de Agroquímica y Tecnología de Alimentos (IATA), Consejo Superior de Investigaciones Científicas (CSIC), Avenida Agustín Escardino 7, 46980 Paterna, Valencia, Spain

**^3^**Department of Organic Chemistry, Faculty of Science and Technology, University of Debrecen, Egyetem tér 1, 4032 Debrecen, Hungary

^4^Institute of Biochemistry, Biological Research Centre of Hungarian Academy of Sciences, Temesvári krt. 62, 6726 Szeged, Hungary

^§^Current address: Sandoz GmbH, Biochemiestrasse 10, 6250 Kundl, Austria

*Corresponding author

**Table S1.** Composition of media used in this study.

| **Description** | **Composition^a^** |
| --- | --- |
| Complete medium (CM) | 2% glucose, 0.2% peptone, 0.1% yeast extract, 0.1% NZ-amine (Sigma), 2% salt solution A, 0,1% trace elements solution (pH 6.5) |
| Salt solution A | 2.6% KCl, 2.6% MgSO_4_ x 7 H_2_O, 7.6% KH_2_PO_4_, 0.2% chloroform |
| *P. chrysogenum* minimal medium (PcMM)^b^ | 2% sucrose, 0.3% NaNO_3_, 0.05% KCl, 0.05% MgSO_4_ x 7 H_2_O, 0.005% FeSO_4_ x 7 H_2_O, 0.1% trace elements solution A, 25 mM NaCl/P_i_ (pH 5.8) |
| Trace elements solution A | 0.1% FeSO_4_ x 7 H_2_O, 0.9% ZnSO_4_ x 7 H_2_O, 0.4% CuSO_4_ x 5 H_2_O, 0.01% MnSO_4_ x H_2_O, 0.01% H_3_BO_3_, 0.01% Na_2_MoO_4_ x 2 H_2_O |
| *P. digitatum* minimal medium (PdMM) | 1% glucose, 0.0001% FeSO_4,_ 0.8% NH_4_NO_3_, 2% salt solution B, 1% trace elements solution B, 40 mM MES buffer (pH 5.3) |
| Salt solution B | 1.5% NaCl, 2.6% MgSO_4_ x 7 H_2_O, 7.6% KH_2_PO_4_ |
| Trace elements solution B | 0.01% H_3_BO_3_, 0.01% CuSO_4_ x 5H_2_O, 0.01% MnSO_4_ x H_2_O, 0.01% Na_2_MoO_4_ x 2H_2_O, 0.01% ZnSO_4_ x 7 H_2_O |

^a^Percent values are given as w/v for solids and v/v for solutions.

^b^For NMR-based analyses, isotope-labelling of PAF[Pc] was performed in PcMM by replacing 0.3% NaNO_3_ with 0.3% Na^15^NO_3_ for single labelling and by replacing 0.3% NaNO_3_ with 0.3% Na^15^NO_3_ and 2% sucrose with 1% ^13^C-glucose (Euriso-Top, Saarbrücken, Germany).

**Table S2.** Oligonucleotides used in this study. Mutation primers for generation of PAF variants are in bold with underlined mismatches for amino acid conversions.

| **Name** | **Sequence 5'-3'** |
| --- | --- |
| M13 | GTAAAACGACGGCCAGTGAG |
| T7var | TACGACTCACTATAGGGCG |
| opaf8 | CAAATCACCACAGTTGCCC |
| opaf9 | TCTCTTCGCTGCAATGGG |
| opaf10 | GCTGCCACCCCCAAGATGACTG |
| **opafF31Nfw** | **GTGCCCCAAGAACGATAACAAG** |
| **opafF31Nrev** | **CTTGTTATCGTTCTTGGGGCAC** |
| **opafY48Qfw** | **GTCGACACCCAGAACAACGC** |
| **opafY48Qrev** | **GCGTTGTTCTGGGTGTCGAC** |
| intron1paf | GTTGTGGTCCCGAACCGTTAGTCG |
| opaf13 | GGTCAAGCCGATTTCGAGAATGACCGTAGTG |
| OJM483 | ATCCCGGGGAATTCAGAGAGCTTTTCGTACG |
| OJM484 | ATTCTAGAGCAGCAGTTTGATAGTTATCCCT |
| 1stF | GGCCCCATGGTGGTAAACAAGTAGTG |
| 1stR | GGTGGCGGCCGCTCTAGAACTAG |
| 2ndF | CACACAGAGCTCATGATCTG |
| 2ndR | GTGTAGGGTAGTAAGCTTCGAG |
| 3rdARsign | CAGGACACCGGCCTC |
| 3rdBF | GACTTCCGCCATTGAATGGTCTCTGCGATCACC |
| 4thFsign | GAGGCCGGTGTCCTGCTTGAGTATAAAGGAG |
| 4thR | CAGAGACCATTCAATGGCGGAAGTC |
| 6thAR | GTAAACCAGATCATGAGCTCTGTGTG |
| 6thBF | CTCGAAGCTTACTACCCTACACCCAC |

**Figure S1**

**pSK275*paf***

GTGGCACTTTTCGGGGAAATGTGCGCGGAACCCCTATTTGTTTATTTTTCTAAATACATTCAAATATGTATCCGCTCATG

AGACAATAACCCTGATAAATGCTTCAATAATATTGAAAAAGGAAGAGTATGAGTATTCAACATTTCCGTGTCGCCCTTAT

TCCCTTTTTTGCGGCATTTTGCCTTCCTGTTTTTGCTCACCCAGAAACGCTGGTGAAAGTAAAAGATGCTGAAGATCAGT

TGGGTGCACGAGTGGGTTACATCGAACTGGATCTCAACAGCGGTAAGATCCTTGAGAGTTTTCGCCCCGAAGAACGTTTT

CCAATGATGAGCACTTTTAAAGTTCTGCTATGTGGCGCGGTATTATCCCGTATTGACGCCGGGCAAGAGCAACTCGGTCG

CCGCATACACTATTCTCAGAATGACTTGGTTGAGTACTCACCAGTCACAGAAAAGCATCTTACGGATGGCATGACAGTAA

GAGAATTATGCAGTGCTGCCATAACCATGAGTGATAACACTGCGGCCAACTTACTTCTGACAACGATCGGAGGACCGAAG

GAGCTAACCGCTTTTTTGCACAACATGGGGGATCATGTAACTCGCCTTGATCGTTGGGAACCGGAGCTGAATGAAGCCAT

ACCAAACGACGAGCGTGACACCACGATGCCTGTAGCAATGGCAACAACGTTGCGCAAACTATTAACTGGCGAACTACTTA

CTCTAGCTTCCCGGCAACAATTAATAGACTGGATGGAGGCGGATAAAGTTGCAGGACCACTTCTGCGCTCGGCCCTTCCG

GCTGGCTGGTTTATTGCTGATAAATCTGGAGCCGGTGAGCGTGGGTCTCGCGGTATCATTGCAGCACTGGGGCCAGATGG

TAAGCCCTCCCGTATCGTAGTTATCTACACGACGGGGAGTCAGGCAACTATGGATGAACGAAATAGACAGATCGCTGAGA

TAGGTGCCTCACTGATTAAGCATTGGTAACTGTCAGACCAAGTTTACTCATATATACTTTAGATTGATTTAAAACTTCAT

TTTTAATTTAAAAGGATCTAGGTGAAGATCCTTTTTGATAATCTCATGACCAAAATCCCTTAACGTGAGTTTTCGTTCCA

CTGAGCGTCAGACCCCGTAGAAAAGATCAAAGGATCTTCTTGAGATCCTTTTTTTCTGCGCGTAATCTGCTGCTTGCAAA

CAAAAAAACCACCGCTACCAGCGGTGGTTTGTTTGCCGGATCAAGAGCTACCAACTCTTTTTCCGAAGGTAACTGGCTTC

AGCAGAGCGCAGATACCAAATACTGTCCTTCTAGTGTAGCCGTAGTTAGGCCACCACTTCAAGAACTCTGTAGCACCGCC

TACATACCTCGCTCTGCTAATCCTGTTACCAGTGGCTGCTGCCAGTGGCGATAAGTCGTGTCTTACCGGGTTGGACTCAA

GACGATAGTTACCGGATAAGGCGCAGCGGTCGGGCTGAACGGGGGGTTCGTGCACACAGCCCAGCTTGGAGCGAACGACC

TACACCGAACTGAGATACCTACAGCGTGAGCTATGAGAAAGCGCCACGCTTCCCGAAGGGAGAAAGGCGGACAGGTATCC

GGTAAGCGGCAGGGTCGGAACAGGAGAGCGCACGAGGGAGCTTCCAGGGGGAAACGCCTGGTATCTTTATAGTCCTGTCG

GGTTTCGCCACCTCTGACTTGAGCGTCGATTTTTGTGATGCTCGTCAGGGGGGCGGAGCCTATGGAAAAACGCCAGCAAC

GCGGCCTTTTTACGGTTCCTGGCCTTTTGCTGGCCTTTTGCTCACATGTTCTTTCCTGCGTTATCCCCTGATTCTGTGGA

TAACCGTATTACCGCCTTTGAGTGAGCTGATACCGCTCGCCGCAGCCGAACGACCGAGCGCAGCGAGTCAGTGAGCGAGG

AAGCGGAAGAGCGCCCAATACGCAAACCGCCTCTCCCCGCGCGTTGGCCGATTCATTAATGCAGCTGGCACGACAGGTTT

CCCGACTGGAAAGCGGGCAGTGAGCGCAACGCAATTAATGTGAGTTAGCTCACTCATTAGGCACCCCAGGCTTTACACTT

TATGCTTCCGGCTCGTATGTTGTGTGGAATTGTGAGCGGATAACAATTTCACACAGGAAACAGCTATGACCATGATTACG

CCAAGCGCGCAATTAACCCTCACTAAAGGGAACAAAAGCTGGGTACCGGGCCCCCCCTCGAGGTCGACGGTATCGATAAG

CTTGATGGCCTAGATGGCCTCTTGCATCTTTGTTTGTATTATACTGTCTTTCTTGTTACACATAATTATTCTAGAATGCC

CCACCGTTACATACGGGACACAGCCATTTACATATGCATGTGGATTACGAGCTAACGAGTTCATTCAAATCTCAGAACTA

TCACATAATCATCATTCCCCTATCGTCAAAGACCGTAAGACAAATCCGGTTCATGCACTGAACCCATTCGGGTAGTGAGT

CATTTACTCAGCACACTCGCGCTGACGCTCGTCGAACACCTTCAATGCCTCCTCGGCAGCCTTGACACCACTGAGAACCA

TGGCACCGAAGGTAGGGCCCATGCGGTTAAAGCCATCAATTTCAGACAGCTCCATACCGCCGATTATCAAGCCCTTAGTA

ACCTCGCGGGTGTTCTTGACGATGGCATCCTCGGCCGAGTTCATGTCGAGACCACGCATGCCACCTAGCTTGTCGACGCT

GCCCATGGACACCAAGCGCTTCGCACAGAAGGCGCCGAATGGCCCATCGTGACCAGTGGTACTGATGATGACAGGAGCGT

TGATAGTGTTGGGGTCCATGCAGGAGTGATCATCGTGGTGAAGGGTGACCAGCGTCCAGTTGACGACAACACCAGCAATC

TGGGGGTTGCCGTTCTCGGTCGGACGGGTGATCAAGTCCTCAACAGCGGTAGCATTGAAGAGCTTGACATTGGGGAAGGA

GAGAACCTTCGACATGAGTGTCGAGGTAAACAGGGAGGCGTGCTTGACGACAACGTAGTTGGGGTTTGCGTCCTCTTCGT

AAGGAACACCCAGCTCGTTCAGGAAGACTTCCGCGGGACGGCGCATGACCATAGCAGAAAAGAGTTGGCCACCCAACCAG

GCACCGCCACCTATTGCACGTTAGTTCCGGAAAGCTGAGTGCAAGGCAATCCATCATGGACTACTGACCAGGAGAGACGC

TGGCCTCGACGATAGCAATCTTCAGGTCCGGACGAGCCTTGGCCAAGACGTACGCAGTGCTCAGACCGCAGGAACCAGCA

CCAACAATGACAACGTCACTTTCAGCGTACTTGTCCAGGTCCTCAAAGTAACGTCTGGTCATGGCACGAGAGACCTGGCT

TTCGCGGATAGGGGCGAACTTGAACTCGTCCCACTTGCCACCGAAATGGTCCAACAGCTTGGTCTGAGAAGCTCCCTCAA

CGGGGACGGTCTCAGAAACCACGACCTTACCCTTGAGGCCGGTAGCGGCCACAGTGGGTTCGTAGATGGCAGCTGGAGGA

GACATGTTTCAAGTTGCAATGACTATCATCTGTTAGCCATTCCATCAACAGGAAGAACGAGAGAAGGCATGACCCTTTTC

GCTGGTATTATCCAGATCAAGTTTTAGCCGTATAATCTCAGAACGAACCCAGTCCATCGATGCCATGTCCTTCTAGACTA

GGATCCTAGAGTCTAGGGCCCAGCTTAGGGAGGGCATGTGAATGCATCGATGACTGGGAACGAACACCGGCCCACGCCAA

AGACGTTACCTAAGATACCTTGATCATTGTGAGAGTCCAGCCAAAAGTATTCCATGACTTCCATCGTATGCCCTCTAGAG

GGCTAATCGAGGAGTGTATTTACATTGTCGGTTGGTTTGGGAACTATAGAAGATGGTCAGTTATTCCAATCACCAAAGGT

TTATCGAAGGGAGGAAGACTTGTTCAGTTTCGTCCGAGGACTTTTGGAATTCAAATCTGAGATAGAGAATTGTGTGGGAT

GAGAGGAAAAGGAAGAAGATGGTGGGGTTCAGAAGGAGGGGTTGAGTTAAATAGCATGGGTTGAGTCAACGTGATAAGGG

CACTATACCGTATAGATCAGCGGCACCCGATTCTATCCGTTCCTTTTGCTCCTCTTTAGCTTTGACCGGTGAGCCGGACA

AGAAACAAGTGAAATCATCCTGACATCGGCGGACGATCTCCTAGCTTTTACATTTCGTTACCAATGGGATCCCGTAATCA

ATTGGCCTGAGTGGCCATCGAATTCCTGCAGGAATTCAGAGAGCTTTTCGTACGAAGTGCGTAATGTACGTAGCATTTTA

TGGTAGCATGCAAAGCACATTTTGCTGCAACCCCAATTTAATGCGGTCCTGCTCAATAATTGATCTGCACTAAGGCCTTG

GCGATGGGGCCAGAAAAGGGTTGTTCAGTGGTGTGTACTCCGTAATGGTCAAGCCGATTTCGAGAATGACCGTAGTGTTC

ATTCATCAGTGCGATATTAAATCAGTTAGCTACTCTATCTGAAAGCTAATAAATTTCTTTACCACTAACAATACTCTTCT

CTGACTGAAAGTACCTTTTCCACTCCCCTCATACTTCATGTTTTAAGCTCAACCGTAGGAAAGCCTGTATATCTTAAAAG

ATTTGGATTTACTCTTCCAGCGCTTACTGTCTGCTCTTTCGGCCGAGCGAACCTTGGCAGTATGATCGGACTATGTACTT

TGTTACACAAAAGGAGAAGCGGGGCTGCCACTGAGGACAACCCCTGTTCAAGGGCTAGCATCCCGCTGTAAGCCCACCCA

TCCCACCTTGAAGTATGCAACTTTTGACCGCCTAGACCATGTGAGCTTATGTTACTGAAATACTACCCGCGAATCATTTC

CTAATTTGCTTTGGCTCGAATCCACCCCAGCCCTACGTAACACAACCGGGAGCTGCCTTACAGCTTGGCTGTATCACAGT

ATCACATAGATACATACATAGTATAGTGCCTTTGCCTTTTCGACCTATAAGCATCCGCCATATGCTAAACCTTCTCATAT

ACCAACATTTTGGATTTGGAGATCATTTCCTAGTGAAACAACTTTATCAAATGCAATGCAGCCATCGTCCTTTGCAGATC

CGAGTGGCCCAGTCACCGTGTCAACGTGTCAGCCGTTTTCTCTGCTTTTTAGGAAATGATTACCACTAGGTAAGCCCAAA

AATATCTTCCTGGTAAACAAGTAGTGCATCTTACCCCGGAGGCTGAAGCAGGTAAGGGATTTTGGAGAGACCCCACCCGT

AAGAATATACCAGCCAAGAGGTCCAGTATCCTGAAGTATGTGAGGCATTAATGTCATTGGAGAAGTCATGCAATCCATAA

GCTGCCACCCCCAAGATGACTGCATTGGACCTGAGCATTGTATGTGTCACCTTTCACACAGAGCTCATGATCTGGTTTAT

AAAGGCGGCTTCATGACCCTCAATTCCATATAGTATCACTCCCATCACAGCATTTCGATATCTTCAACCACTTTAACCTT

CTCCAGAGGATCATCATCTCAAGCCCTTCATA**ATGCAAATCACCACAGTTGCCCTTTTTCTCTTCGCTGCAATGGGCGGG**

**GTAGCCACCCCCATTGAGTCTGTATCAAACGACCTCGATGCCAGGGCTGAGGCCGGTGTCCTGGCCAAATACACCGGAGT**

**GAGTAAACATCAATATCCCATACCTTAAGTACTCACTTGGGAATCGCGACTAACGGTTCGGGACCACAACTCAGAAATGC**

**ACCAAATCTAAGAACGAATGTAAATACAAGAACGATGCTGGAAAGGACACTTTTATCAAGTGCCCCAAGTTTGATAACAA**

**GAAGGTAGAATATCAATCATTCGGAAGTAGCCATCTGAATCGATTTCGTGCTAATCTCGCTCTTTTTTCCAGTGCACCAA**

**GGATAATAACAAATGTACCGTCGACACCTACAACAACGCTGTCGATTGTGACTAG**ATGGTCTCTGCGATCACCAGGGCAT

TTAATGGTTTTTGGTTCCCTTCTTGTTGGTGATATGCGAGATGCCCTGTGATTCTCGAAGCTTACTACCCTACACCCACA

AGGACTCGAACCAAGGACTGCTCGGTGGGTGATACATATACACCCAGTATCTATCCAGCTTCAATTTTCGGCGAATTTTG

TTTCTTATTTCATAAAGACACTCGTTTGATATCTAGCTAGATATTGTTGCTCATCAACGAAATGGTTGTAGATTATCGAA

TATATCCAGCATCTTTTGATCGTAGTCGGAAGTGAAATGGAGTACTATGATACGACACATGTACATTGTAAGCAGAAATA

GGCTAGAGGGATAACTATCAAACTGCTGCAGCCCGGGGGATCCACTAGTTCTAGAGCGGCCGCCACCGCGGTGGAGCTCC

AATTCGCCCTATAGTGAGTCGTATTACGCGCGCTCACTGGCCGTCGTTTTACAACGTCGTGACTGGGAAAACCCTGGCGT

TACCCAACTTAATCGCCTTGCAGCACATCCCCCTTTCGCCAGCTGGCGTAATAGCGAAGAGGCCCGCACCGATCGCCCTT

CCCAACAGTTGCGCAGCCTGAATGGCGAATGGGACGCGCCCTGTAGCGGCGCATTAAGCGCGGCGGGTGTGGTGGTTACG

CGCAGCGTGACCGCTACACTTGCCAGCGCCCTAGCGCCCGCTCCTTTCGCTTTCTTCCCTTCCTTTCTCGCCACGTTCGC

CGGCTTTCCCCGTCAAGCTCTAAATCGGGGGCTCCCTTTAGGGTTCCGATTTAGTGCTTTACGGCACCTCGACCCCAAAA

AACTTGATTAGGGTGATGGTTCACGTAGTGGGCCATCGCCCTGATAGACGGTTTTTCGCCCTTTGACGTTGGAGTCCACG

TTCTTTAATAGTGGACTCTTGTTCCAAACTGGAACAACACTCAACCCTATCTCGGTCTATTCTTTTGATTTATAAGGGAT

TTTGCCGATTTCGGCCTATTGGTTAAAAAATGAGCTGATTTAACAAAAATTTAACGCGAATTTTAACAAAATATTAACGC

TTACAATTTAG

**Figure S1.** Nucleotide sequence of the plasmid pSK275*paf* (7051 bp) containing the *paf* gene expression cassette with 5'-UTR (blue, nt 4272-5552), *paf* gene (magenta, nt 5553-5975) and 3'-UTR (orange, nt 5976-6349. The *ampR* (nt 327-986) and *ptrA* (nt 2247-4243) resistance genes are marked in brown and green, respectively.

**Construction of pSK275*nfap^paf_signal^***

The *paf* gene with partial 5’- and 3’-UTR from pSK275*paf* was amplified and cloned into *Nco*I/*Not*I digested pGEM-T (pGEM-T_1ststep) (Fig. S2A). In the second step the *paf* gene with partial 5’- and 3’-UTR from pGEM-T_1ststep was amplified and cloned into *Sac*I/*Hind*III digested pUC19 (pUC19_2ndstep) (Fig. S2B). In the third step partial *paf* 5’-UTR with the PAF pre-pro sequenece encoding region and partial *paf* 3’-UTR with the last 15 nucleotides (GACTTCCGCCATTGA) of *nfap* ORF was amplified from pUC19_2ndstep (Fig. S2C). Then the mature NFAP encoding cDNA with the last 15 nucleotides (GAGGCCGGTGTCCTG) of the PAF pre-pro sequence and partial *paf* 3’-UTR was amplified from the previously constructed pBSK(-)*nfap* (Virágh et al. 2014, Protein Expr Purif; 94:79-84.) containing the cDNA of the mature NFAP (Fig. S2D). Joint PCR was used to assemble partial *paf* 5’-UTR with the PAF pre-pro sequenece encoding region, partial *paf* 3’-UTR with last 15 nucleotides (GACTTCCGCCATTGA) of *nfap* ORF, and mature NFAP encoding cDNA with the last 15 nucleotides (GAGGCCGGTGTCCTG) of the PAF pre-pro sequence and partial *paf* 3’-UTR. Then this construct was cloned into *Sac*I/*Hind*III digested pUC19 (pUC19_5thstep) (Fig. S2E). In the next steps partial *paf* 5’- and 3’-UTRs, and mature NFAP encoding cDNA with partial *paf* 5’-UTR, PAF pre-pro sequence encoding region and partial *paf* 3’-UTR were amplifed from pGEM-T_1ststep and pUC19_5thstep, respectively. Finally these amplicons were assembled by joint PCR (Fig. S2F) and cloned into *Bsp*MI/*Not*I digested pSK275*paf* (pSK275*nfap^paf_signal^*).

**A**


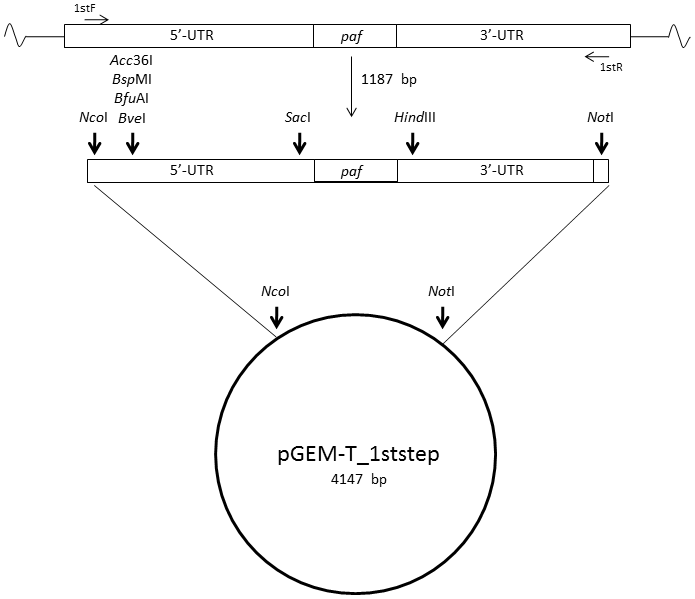


**B**


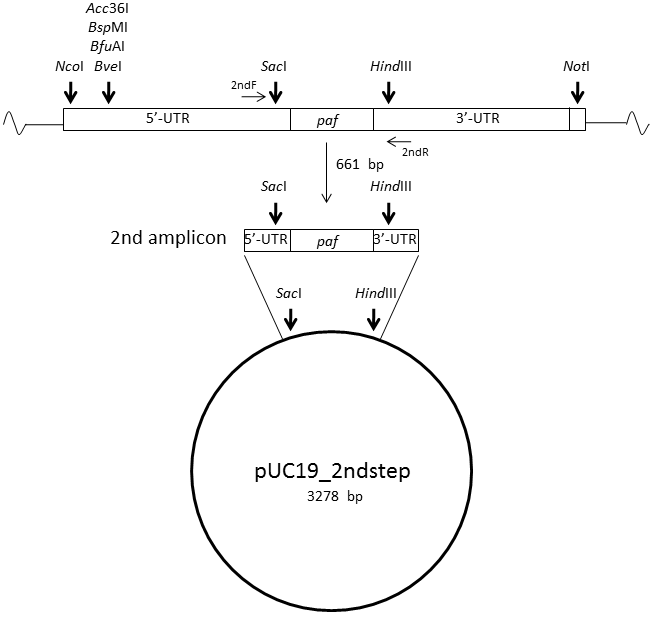


**C**


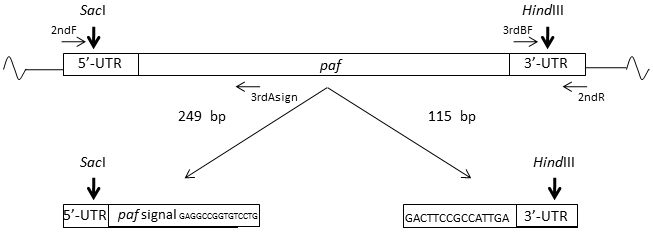


**D**


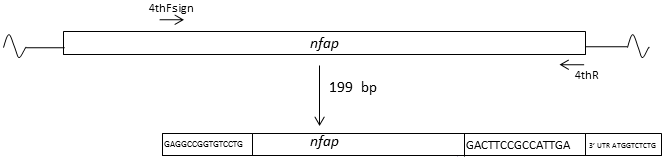


**E**


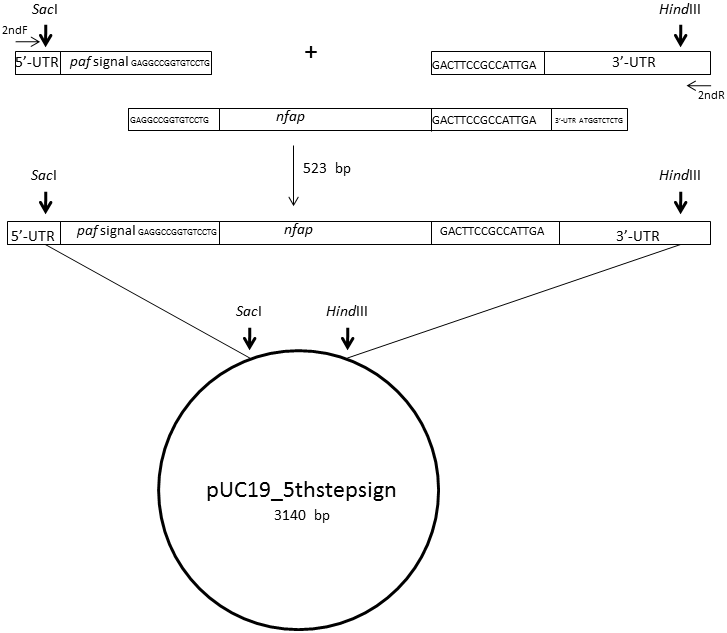


**F**


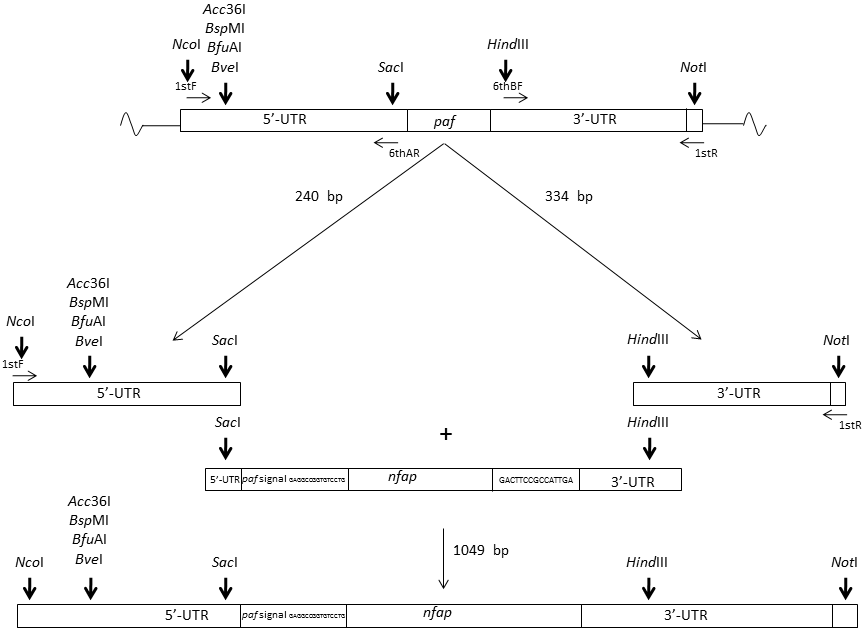


**Figure S2.** Schematic representation of the cloning strategy of the NFAP expression vector pSK275*nfap^paf_signal^*. The restriction sites are shown with vertical bold arrows; the oligonucleotides for PCR amplifications are indicated by horizontal light arrows.

**Southern blot analysis**

In Southern blotting experiments the random integration of the transforming DNA into the fungal genomes was proved by using a 1.3 kb DIG-labelled PCR probe spanning a part of the *paf* gene and the *paf* promoter (Fig. S3). The digestion of genomic DNA of the recipient strain *P. chrysogenum* *∆paf* with *Nde*I resulted in two hybridizing fragments sized 3.4 kbp and 9 kbp, or 1.3 kbp and 3.1 kbp, when the restriction enzymes *Nhe*I/*Xho*I were used (Supplements, Fig. S3A). The 3.4 kbp and 9 kbp fragments were found in the *Nde*I-digested genomic DNA of *P. chrysogenum* strains PAF^Y48Q^, PAF^F31N^ and PAF (Fig. S3B), whereas the 1.3 kbp and 3.1 kbp fragments were visible in the *Nhe*I/*Xho*I-digested genomic DNA of *P. chrysogenum* strain NFAP (Fig. S3C). This indicated that the nourseothricin resistant gene (*nat1*) cassette was still present in the genomes of these transformed strains. The hybridizing fragments of 2.7 kbp in size proved the presence the plasmids with the expression cassettes in the genomes of *P. chrysogenum* strains PAF^Y48Q^ (Fig. S3B (a)), PAF^F31N^ (Fig. S3B (b)) and PAF (Fig. S3B (c)). In the *P. chrysogenum* strain NFAP the integration of the expression plasmid into the genome was evident from the 2.5 kbp sized fragment (Fig. S3C). The additional signals of various sizes and intensities further proved that at least single or multiple-copy random plasmid integrations in these *P. chrysogenum* strains had occurred. The analysis of the *Nde*I digested genomic DNA of *P. digitatum* strain PAF revealed the presence of at least three copies of the *paf* expression cassette (Fig. S3D (b)) in contrast to the lack of signals in the not transformed *P. digitatum* recipient strain (Fig. S3D (a)).

**Figure S3**


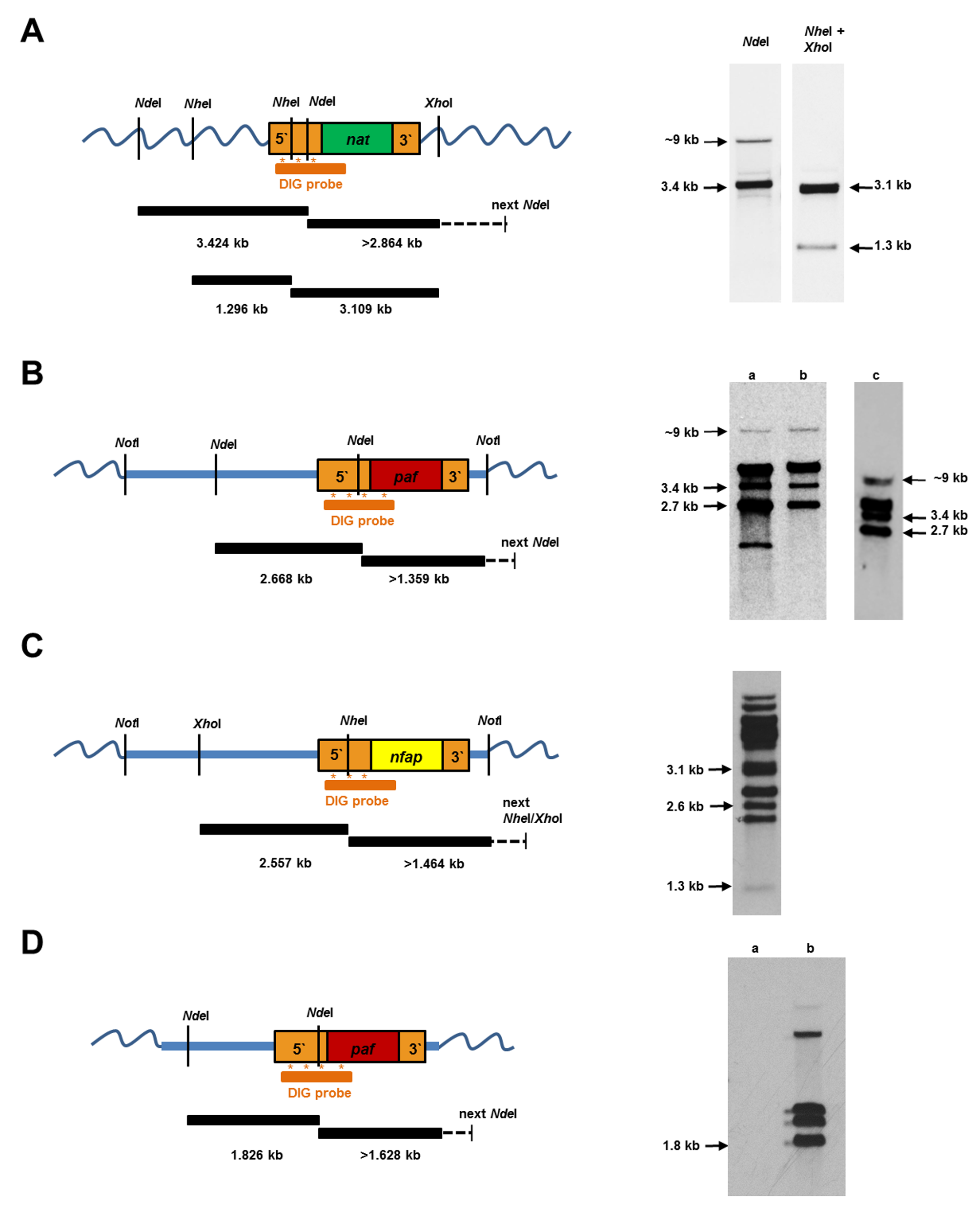


**Figure S3.** Southern blot analysis for genomic integration of the *P. chrysogenum* expression cassette in the *Penicillium* cell factories. Left: schematic representation of the expression cassettes comprising the *paf*-specific 5'-UTR and 3'-UTR (orange) and the respective gene coding sequences (green: *nat1*; red: *paf*; yellow: *nfap*). The pSK275 plasmid backbones and the *Penicillium* sp. genomes are represented by blue straight and wavy lines, respectively. The position of the *paf* 5´-UTR-specific DIG-labelled probe (orange line) is indicated with asterisks representing the region of hybridization, the hybridizing fragments with the expected sizes are delineated below in black. Right: Southern blots of digested genomic DNA showing site-specific and random genomic integrations (RI). (A) Recipient strain *P. chrysogenum* *∆paf*, digested with *Nde*I or with *Nhe*I/*Xho*I (control). (B) Transformed *P. chrysogenum* *∆paf* strains for the production of (a) PAF^Y48Q^, (b) PAF^F31N^ and (c) PAF, DNA digested with *Nde*I. (C) Transformed *P. chrysogenum* *∆paf* strain for NFAP production, DNA digested with *Nhe*I/*Xho*I and (D) *P. digitatum* (a) not transformed and (b) transformed for PAF production, DNA digested with *Nde*I.

**Figure S4**


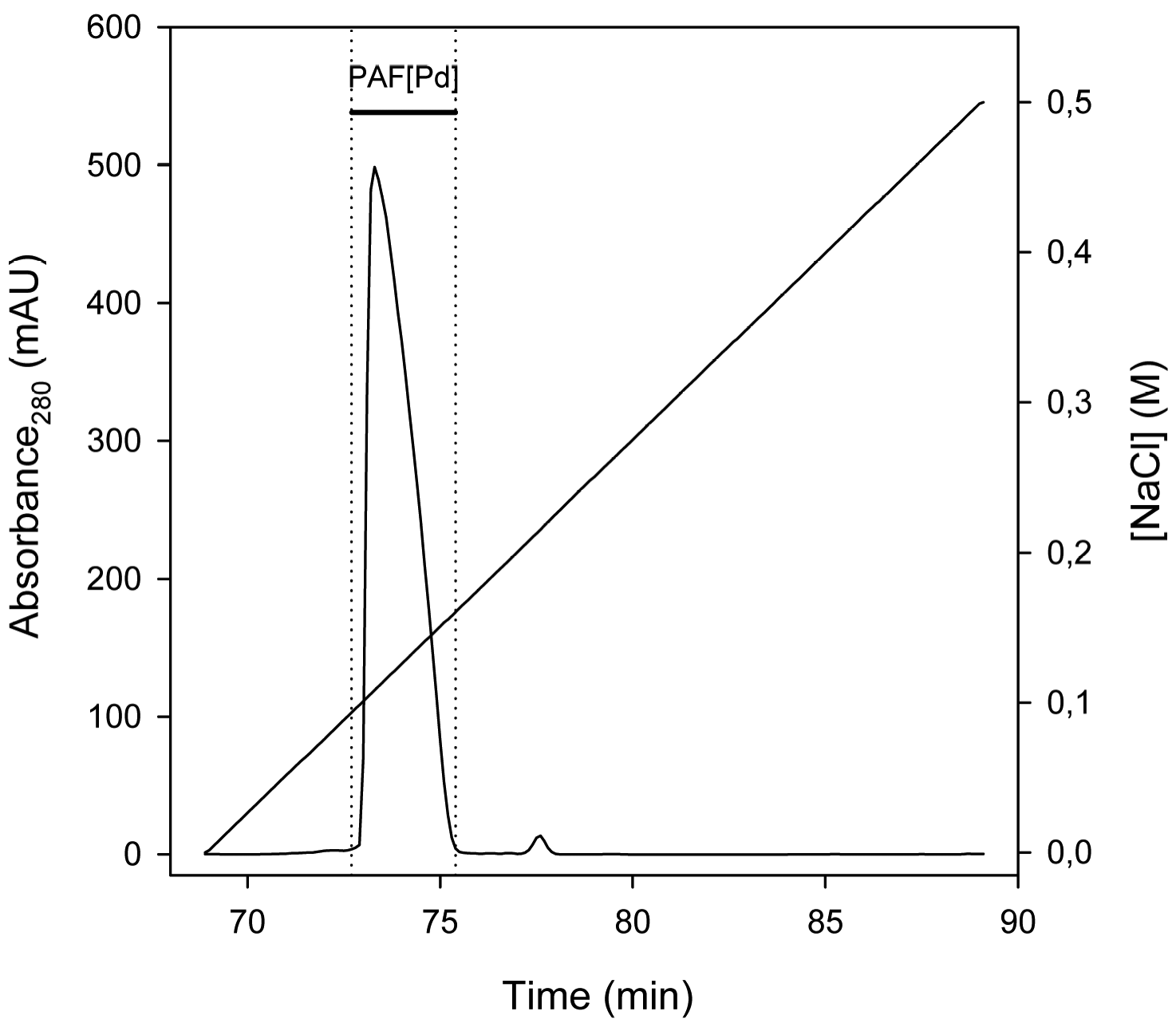


**Figure S4.** The elution pattern of PAF[Pd] purified by cation-exchange chromatography. Experimental details are described in the text.

**Figure S5**


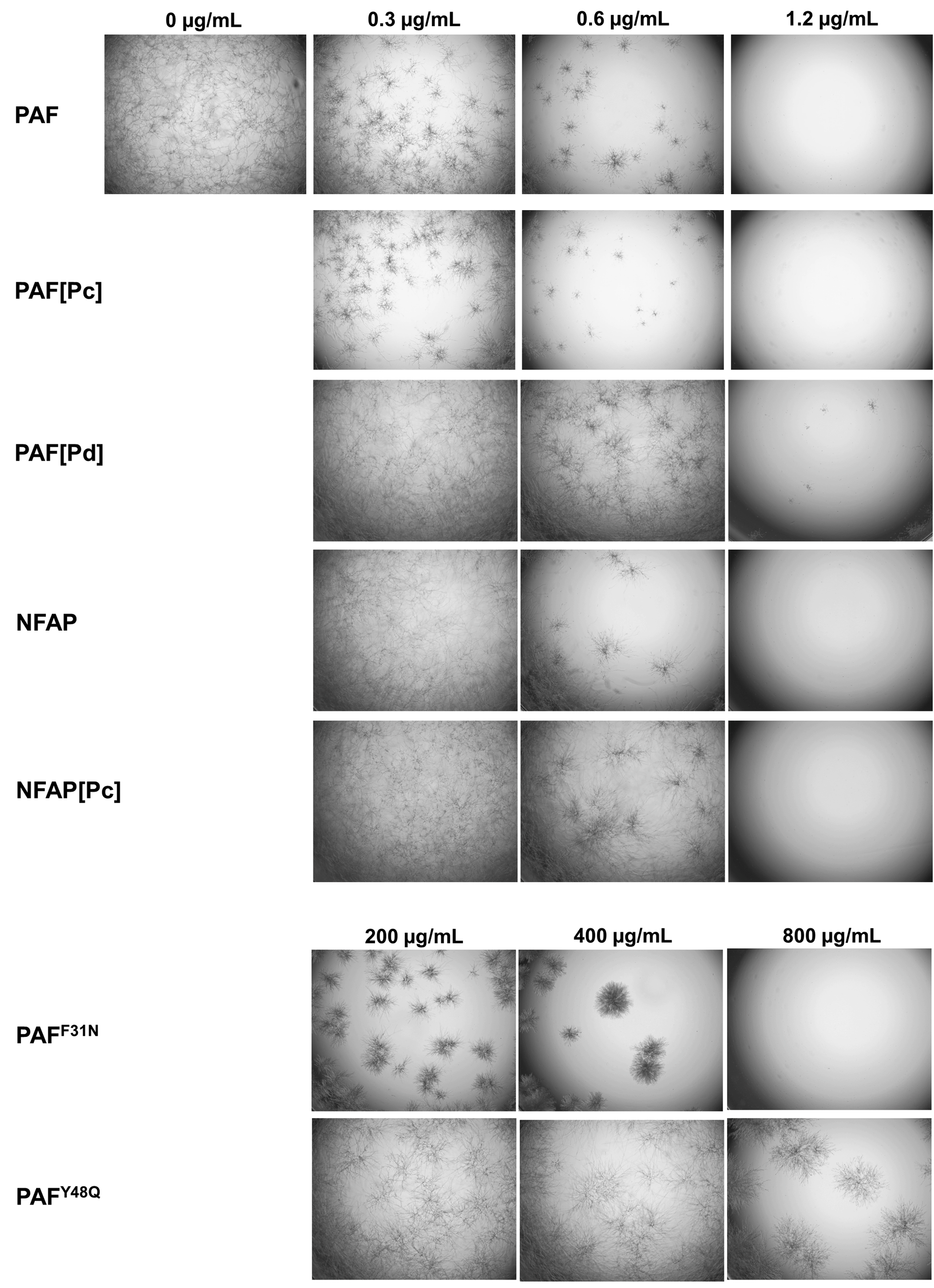


**Figure S5.** Growth inhibition assays with *A. niger* exposed to 0-1.2 μg/mL of PAF, PAF[Pc], PAF[Pd], NFAP, NFAP[Pc], and 0-800 μg/mL of PAF^F31N^ and PAF^Y48Q^. Overview images were taken after 48 h of incubation at 30 °C. One representative experiment out of three is presented.
